# MEDA-PKD: a dynamic, real-time analytics platform for precision care in autosomal dominant polycystic kidney disease

**DOI:** 10.1038/s41746-026-03267-0

**Published:** 2026-10-20

**Authors:** Franziska Grundmann, Sita Arjune, Samer Alkarkoukly, Malte P. Bartram, Bodo B. Beck, Thomas Benzing, Volker Boehm, Katrin Bohl, Volker Burst, Martin Busch, Figen Cakiroglu, Sadrija Cukoski, Peter Gerke, Nils Große-Hokamp, Jan Halbritter, Ludwig Matrisch, Thorsten Persigehl, Friederike Petzold, Johannes Schmidt, Roland Schmitt, Polina Todorova, Christoph Wanner, Philipp Antczak, Roman-Ulrich Müller

**Affiliations:** 1 Department II of Internal Medicine and Center for Molecular Medicine Cologne, University of Cologne, Faculty of Medicine and University Hospital Cologne, Cologne, Germany; 2 Cologne Excellence Cluster on Cellular Stress Responses in Aging-Associated Diseases (CECAD), University of Cologne, Faculty of Medicine and University Hospital Cologne, Cologne, Germany; 3 Center for Rare Diseases Cologne, Faculty of Medicine and University Hospital Cologne, University of Cologne, Cologne, Germany; 4 Center of Excellence in Nephrology, University Hospitals Aachen, Cologne, Düsseldorf, North Rhine-Westphalia, Germany; 5 Institute for Biomedical Informatics, University of Cologne, Faculty of Medicine and University Hospital Cologne, Cologne, Germany; 6 Institute of Human Genetics, University of Cologne Faculty of Medicine and University Hospital Cologne, Würzburg, Germany; 7 Institute for Genetics, University of Cologne, Cologne, Germany; 8 Department of Nephrology, Medical Faculty, University Hospital Düsseldorf, Heinrich-Heine-University Düsseldorf, Düsseldorf, Germany; 9 Emergency Department, University of Cologne, Faculty of Medicine and University Hospital Cologne, Cologne, Germany; 10 Department of Internal Medicine III, University Hospital Jena, Friedrich-Schiller-University, Jena, Germany; 11 Department of Internal Medicine I, University Hospital Schleswig-Holstein, Campus Lübeck, Lübeck, Germany; 12 Nierenzentrum Lübeck, Lübeck, Germany; 13 Institute for Diagnostic and Interventional Radiology, University of Cologne, Faculty of Medicine and University Hospital Cologne, Cologne, Germany; 14 Department of Nephrology and Medical Intensive Care, Charité – Universitätsmedizin Berlin, Berlin, Germany; 15 Renal Division, Department of Medicine, Faculty of Medicine and Medical Center, University of Freiburg, Freiburg, Germany; 16 Division of Nephrology, Department of Endocrinology, Nephrology, and Rheumatology, University of Leipzig Medical Center, Leipzig, Germany; 17 Bonacci GmbH, Cologne, Germany; 18 Department of Nephrology and Hypertension, Hannover Medical School, Hannover, Germany; 19 Department of Nephrology and Hypertension, University Hospital Schleswig-Holstein, Kiel, Germany; 20 Center for Rare Diseases Düsseldorf, Medical Faculty, University Hospital Düsseldorf, Heinrich-Heine-University Düsseldorf, Düsseldorf, Germany; 21 Department of Clinical Studies and Epidemiology, Comprehensive Heart Failure Center, University Hospital Würzburg, Würzburg, Germany; 22 Robert Bosch Hospital, Stuttgart, Germany; 23 Institute of Diagnostic and Interventional Radiology, University Hospital Würzburg, Würzburg, Germany; 24 Nephrologicum Markgräflerland MVZ GmbH, Müllheim, Germany; 25 Department I of Internal Medicine, University of Cologne, Faculty of Medicine and University Hospital Cologne, Cologne, Germany; 26 Department III of Internal Medicine, Heart Center, University of Cologne, Faculty of Medicine and University Hospital Cologne, Cologne, Germany; 27 Praxisgemeinschaft Dr. Peschel, Leipzig, Germany

**Keywords:** Computational biology and bioinformatics, Diseases, Medical research, Nephrology

## Abstract

The use of medical real-world data is constrained by fragmented data infrastructures and the absence of tools integrating longitudinal clinical with molecular data at scale. Autosomal dominant polycystic kidney disease (ADPKD), the most common monogenic cause of kidney failure, exemplifies this gap given its phenotypic complexity and the need for individualized treatment decisions. We present MEDA-PKD (MEdical DAta translation service of Polycystic Kidney Disease), an interactive, near-real-time analytics platform that integrates clinical routine with research data from the German AD(H)PKD Tolvaptan Registry (NCT02497521, DRKS00008910) into a harmonized database (https://doi.org/10.18716/adpkd_registry). MEDA-PKD provides automated near-real-time analysis and dynamic visualization, replacing traditional static approaches to clinical cohorts. As of March 2026, MEDA-PKD visualized data from 1735 patients (mean follow-up 796 days). eGFR slope analysis of data from patients initiating tolvaptan therapy (332 patients) demonstrates the platform’s capacity to evaluate real-world therapeutic impact in real-time. Other tabs include family history, medication, lifestyle, quality of life and a wide array of lab values (20,894 entries). Besides, MEDA-PKD integrates proteomics data and allows for secure individual patient-level data access. MEDA-PKD represents a paradigm shift by transforming static cohort data into a living, actionable knowledge resource in ADPKD and will serve as a template for other diseases.

## Introduction

Chronic non-communicable diseases (NCD) are a major burden to society, patients, and their families. Chronic kidney disease (CKD) is a key example for these challenges. Autosomal dominant polycystic kidney disease (ADPKD) is the most common monogenic cause of CKD and kidney failure^1,2^. Formation of cysts begins in childhood, progresses over life-time and eventually replaces functional parenchyma leading to loss of kidney function. Kidney failure - which occurs by the sixth decade of life in about 50% of the patients - comes with a major burden both to ADPKD patients and healthcare systems^2,3^. Approximately 10% of all patients requiring kidney replacement therapy, i.e., kidney transplant or dialysis, have ADPKD as the underlying cause. Besides loss of kidney function, organ growth leads to various symptoms with high relevance to quality-of-life including pain and abdominal fullness^4^. As a systemic disorder, ADPKD also causes cyst formation in other organs, notably the liver with ~10–20% of the affected individuals presenting symptomatic polycystic liver disease (PLD)^5,6^. The disease also comes with an increased incidence of cerebral aneurysms (~8–12%), colonic diverticula, and cardiac valve defects^7^.

More than 90% of all ADPKD cases are caused by pathogenic variants in two genes – *PKD1* and *PKD2*^8^. Despite its genetic nature, clinical outcomes in ADPKD including kidney failure show a high degree of intrafamilial variability^9^ and cannot be accurately predicted yet. However, prediction of clinically relevant outcomes is of special importance in ADPKD to optimize patient counseling, implement individualized disease management and allow patients to determine the answers to major life questions. The only treatment specifically approved for ADPKD, the vasopressin-V2-receptor antagonist tolvaptan^10^, comes with relevant side effects including a risk of liver damage and severe polyuria (up to 15 L/day)^11^. In addition, while tolvaptan slows loss of kidney function by about 25%^12,13^, it will not prevent kidney failure in most cases. As to extrarenal manifestations, there are no pharmacological therapeutic options to date.

Consequently, a better characterization of the disease in a real-time fashion using large cohorts would be an important step to allow for (1) informing patients, physicians and other stakeholders about in-depth disease characteristics, (2) examining the impact of therapeutic interventions in a real-life setting, (3) leveraging the integration of clinical routine data with research data for the optimization of outcome prediction, (4) building the foundation for improved individual patient-counseling in the future and (5) allowing for direct patient stratification, e.g., as a basis for clinical trials.

Digital health infrastructures^14^, the FAIR data principles^15^, and the learning-health-system concept^16^ together provide the conceptual and technical foundation that makes near-real-time, multi-source cohort platforms like MEDA-PKD feasible and clinically useful. Modern digital-health approaches emphasize continuous capture, integration and reuse of routine clinical data (including EHRs and laboratory feeds) to support rapid knowledge generation and practice change, rather than isolated, static analyses. FAIRification of clinical and research data improves findability, interoperability and reusability across institutions, enabling federated analyses and safer data sharing while preserving local control^17^, capabilities that MEDA-PKD was designed to leverage. Finally, embedding MEDA-PKD within a learning health system workflow closes the loop from data capture to knowledge generation and back into care, supporting iterative evaluation of interventions such as tolvaptan in real-world settings and accelerating translation of discoveries into improved patient management.

The usual approach to this topic is based on databases lacking standardization coupled to data cleaning and analysis in the context of individual projects. This approach results in static publication of visualized data allowing insight at a single-point in time and usually addressing a highly limited number of data points contained in the original list of variables collected. Furthermore, current standards usually require the manual transfer of data from clinical routine systems to research databases, a highly error-prone system further limiting the number of variables available to the analyses. However, thousands of variables are contained in clinical data systems and partly, e.g., for lab values, are available in structured databases already. MEDA-PKD (MEdical DAta translation service applied to Polycystic Kidney Disease) addresses these challenges employing ADPKD as a highly relevant medical use-case. To reach this goal, MEDA-PKD integrates numerous data sources including direct access to laboratory values from the clinical database. Leveraging a PostgreSQL backend and R/Shiny-powered front-end visualization, MEDA-PKD provides clinicians and researchers with continuously updated insights into patient trajectories across key domains including eGFR dynamics, imaging phenotypes, extrarenal manifestations, medication use, quality of life, and family history. Built on a modular, containerized architecture across the R/Shiny app as well as using Docker and Jenkins to perform underlying database updates, MEDA-PKD ensures long-term scalability, reproducibility, and interoperability. The system supports seamless integration of external datasets, enabling federated comparisons while preserving local data ownership.

MEDA-PKD builds on the generic MEDA framework, a Python/PostgreSQL-based universal translation service for clinical and research data initially described by Schmidt et al.^18^. The MEDA approach results in dynamic dashboards updating all analyses automatically after publication to provide the field with a near-real-time view into the characteristics of the cohort. Importantly, the dashboards already unify clinical data with omics data in a first pilot approach. Users can filter for patient characteristics such as age, sex, tolvaptan usage and Mayo Imaging Class (MIC) within the individual dashboard to allow for an active user-webtool interaction for improved usability regarding individual research questions.

Taken together, MEDA-PKD is an important step forward for medical research in ADPKD. The data dictionary underlying MEDA-PKD will be provided to other centers to allow for extension to additional cohorts. Importantly, this data infrastructure is not limited to ADPKD but can essentially be employed for any disease setting after adaptation of the clinical variables required and definition of the research questions to design disease-specific dashboards.

## Results

### Baseline characteristics of the AD(H)PKD cohort

**Fig. 1:**
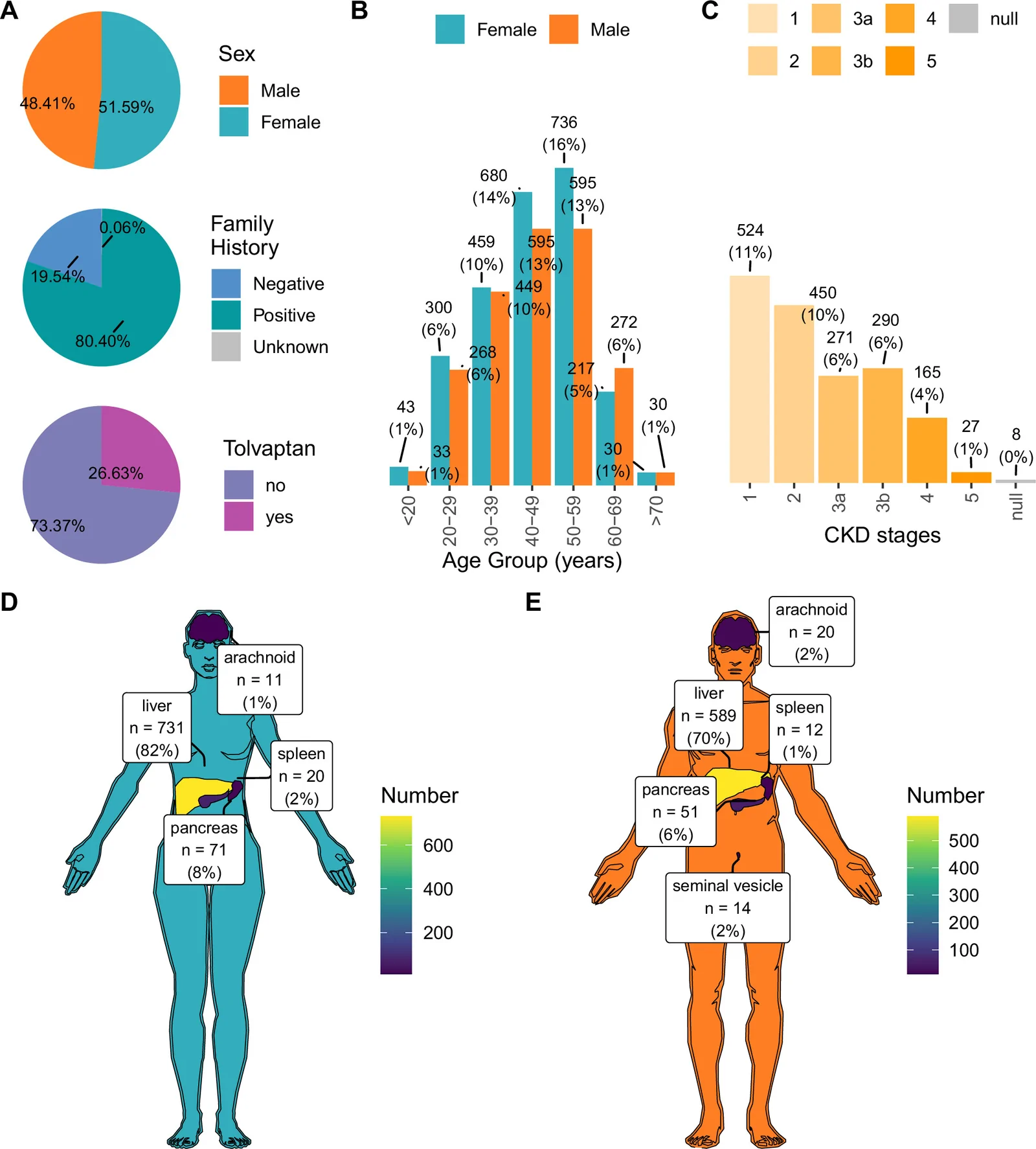
Dashboard “Baseline Characteristics” and “Extrarenal Manifestations”. **A** Fraction of total graphs showing sex, family history as well as percentage of patients on tolvaptan at any point during data collection. **B** Age distribution by sex at baseline. **C** CKD eGFR stages at baseline. **D** Extrarenal cysts in female patients. **E** Extrarenal cysts in male patients.

The technical backbone of the MEDA platform has recently been published by our group^18^ and is now applied to ADPKD as a clinically relevant use-case with a defined need for real-life data analysis. The specific set of data sources fed into MEDA for this use-case includes research databases, the clinical information system ORBIS and omics data (for an overview see Fig. S1). The figures provided in the main body of this manuscript depict the static view on the visualizations as of March 3rd, 2026. MEDA-PKD itself is accessible at https://doi.org/10.18716/adpkd_registry and a first introduction to the platform is provided as a screencast video on the welcome page of this site (https://doi.org/10.18716/cecad/adpkd_1080p). MEDA-PKD provides data visualizations, which are updated automatically on a daily basis and the resulting graphs are provided in 13 dashboards dedicated to individual topics of interest (Fig. S2A). Data entering the graphs can be filtered by the user to obtain an insight into clinically relevant subgroups (e.g., age, sex, tolvaptan usage, MIC, Fig. S2B). The AD(H)PKD cohort study was set up in 2015 to further characterize natural disease course and the impact of the newly approved targeted therapeutic tolvaptan. AD(H)PKD recruits patients with ADPKD, confirmed by genetics or imaging criteria, with an eGFR ≥15 ml/min/1.73 m^2^. As of March 3rd, 2026, the study had recruited 1750 patients with sufficient data availability for MEDA-PKD in 1735 patients, 52% of whom were female and 48% male (Table 1, Fig. 1A, Fig. S3, Fig. S4A). 80.4% of patients reported a positive family history (Fig. 1A, Fig. S5). Reported median age was 44 years at baseline (Table 1, Fig. S3). 7% were already on tolvaptan at baseline and 26% reported being on or having taken tolvaptan at any of the visits (Table 1, Fig. 1 A, Fig. S3). Median age at diagnosis was 27 years and mean GFR was 69 ml/min/1.73 m^2^ (Table 1, Fig. 1B, Figs. S3, S4B). 12% and 4% reported a very early onset of dialysis or kidney transplantation before the age of 50, respectively, in the affected parent (Fig. S5). First time data loading times range between 673 ms and 3 s depending on the dashboard highlighting the dynamic nature of MEDA-PKD. Data completeness is >90% for most variables excluding aspects which need active monitoring outside routine care (e.g., intracerebral aneurysms (45%)), require a minimum number of longitudinal values (e.g., eGFR slope (57%)) or are calculated as composite scores (e.g., PROPKD score (25%)) (Table S1).

**Table 1:** Baseline characteristics of the AD(H)PKD cohort (March 3rd, 2026: *N* = 1735)

	Mean	SD	Median	Min	Max	Missing
Age (years)	43.8	12.8	44.0	18.0	83.0	
Age at Diagnosis (years)	28.0	13.8	27.0	0.0	83.0	73 (4.2%)
Weight (kg)	82.7	19.9	80.0	40.0	189.0	26 (1.5%)
Height (cm)	175.8	10.3	175.0	107.0	209.0	23 (1.3%)
Systolic BP (mmHg)	138.0	17.3	136.0	71.0	228.0	56 (3.2%)
Diastolic BP (mmHg)	86.9	11.8	86.0	33.0	144.0	56 (3.2%)
eGFR (ml/min/1.73 m^2^)	69.3	31.9	67.2	5.6	143.0	24 (1.4%)
htTKV (ml/m)	1010	843	767	132	9870	176 (10.1%)
	**Female (%)**		**Male (%)**			
Sex	895 (51.6%)		840 (48.4%)			

### Extrarenal manifestations

Patients were also asked about or examined for key extrarenal manifestations. It is important to note that, in a real-life study setting and in line with the KDIGO guideline, no routine screening, e.g., for intracranial aneurysms was performed. The vast majority of patients showed liver cysts (82% in female and 70% in male patients, Fig. 1D, E). Arachnoid (1% in female and 2% in male patients), splenic (2% in female and 1% in male patients), pancreatic (8% in female and 6% in male patients) and seminal vesicle cysts (2% in male patients) were reported at a much lower prevalence (Fig. 1D, E). A large proportion of patients showed cardiac valve abnormalities, the vast majority of which were mild in nature. Mitral regurgitation was the most common finding affecting 21% in female and 19% in male patients (Fig. S4C). Intracranial aneurysms had been detected in ~2% of male and female patients and hemorrhage had occurred in ~1%, again without differences between male and female participants (Fig. S4D).

### General medication usage and lifestyle

78% of participants were on at least one antihypertensive drug. ACE-inhibitors or AT1 receptor antagonists were the most commonly used drug classes for this purpose (Fig. S6A). 2% of patients reported regular intake of non-opioid pain medication and 1% reported opioid usage for pain control (Fig. S6B). As to lipid lowering drugs, 14% of participants were on a statin as long-term medication (Fig. S6C). 19% of patients reported to be active-smokers with 33% reporting <20 pack years, 8% 20–40 pack years and 2% ≥40 pack years (Fig. S7A, B). 12% of participants did not drink any coffee. 56% drank an equivalent of 1–3 cups per day and 33% reported a coffee consumption of >3 cups a day (Fig. S7C). Alcohol consumption was reported by 71% with only 4% consuming ≥25 g of alcohol per day (Fig. S7D).

### Predictors of disease progression

**Fig. 2:**
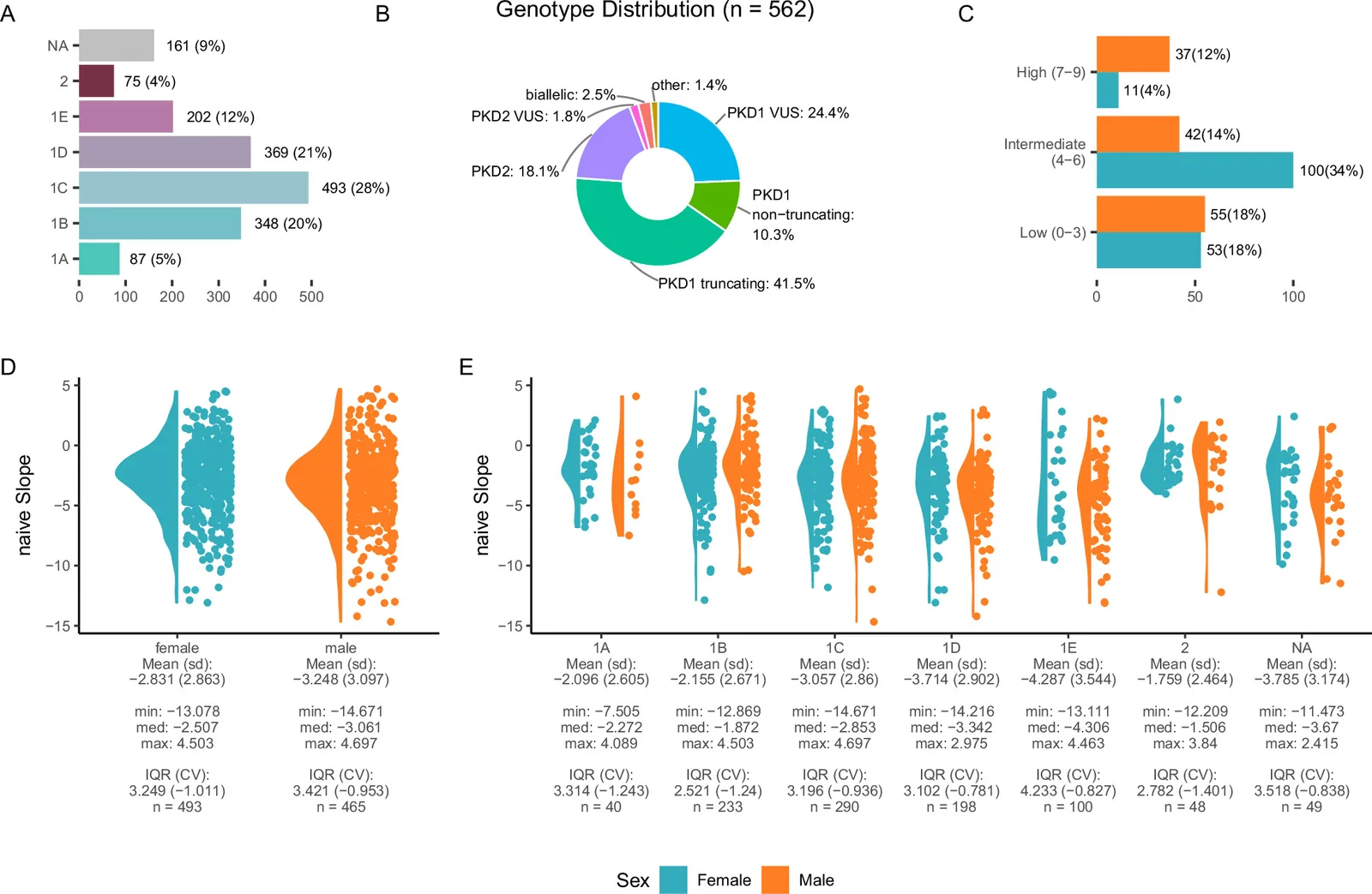
Dashboard “Predictors of disease progression” and “Kidney function decline”. **A** Mayo Imaging Classification across the entire cohort. **B** Pathogenic and likely pathogenic variants classified as PKD1 truncating, PKD1 non-truncating, PKD2 and other (genes known to be causal in ADPKD other than PKD1 and PKD2). Variants of uncertain significance (VUS) in PKD1 and PKD2 are reported separately. Patients showing at least one pathogenic or likely pathogenic variant AND a second variant in an ADPKD-gene which is at least a VUS are reported as biallelic. (Likely) benign variants are not reported. **C** PROPKD Score grouped by high, intermediate and low risk of rapid progression including data only from patients who have reached the age of 35 years and for whom genotype data is available. For the complete dashboards see Fig. S8 and online tool. **D** eGFR slope (in ml/min/1.73 m²/yr) of male and female patients while not being on tolvaptan. **E** eGFR slope (in ml/min/1.73 m²/yr) of male and female patients by Mayo Imaging Class while not being on tolvaptan.

As expected, total kidney volume increased with patient age and male patients showed larger kidneys than female patients (Fig. S8A). MIC was available for >90% of participants (Fig. 2A). 61% of patients for whom a volumetry was available (*n* = 1574) showed a MIC indicating rapid progression (1C-1E) and 4% had been classified as showing atypical cyst distribution/morphology, i.e., MIC 2 (Fig. 2A). 41.5% of those with an available genotype (*n* = 562) showed a truncating PKD1 variant, 10.3% a non-truncating PKD1 variant and 18.1% a variant in the PKD2 gene (counting ACMG class 4 or 5 variants only; Fig. 2B), while the remainder contained minor PKD genes as well as variants of uncertain significance and biallelic cases. 65% of the entire cohort had experienced urological complications as defined in the PROPKD score (Fig. S8B) and among patients ≥35 years of age these had first manifested before the age of 35 in 16% (Fig. S8D). Flank pain was the most common urological complication followed by macrohematuria (Fig. S8F). Arterial hypertension was known in 84% of participants (Fig. S8C) and, in patients > 35 years of age, had first been diagnosed before the age of 35 in 24% (Fig. S8E). Based on these data, the PROPKD score could be calculated for 298 patients (i.e., genotype available and age ≥35 years) and indicated intermediate or high risk of rapid progression in 47% and 16% of participants, respectively, with male patients being more likely to be in the high-risk group (Fig. 2C).

### Kidney function trajectories

As of March 3rd, 2026, 18,841 creatinine values were available for the AD(H)PKD cohort. Since tolvaptan as the only available disease-modifying treatment is known to have a clear impact on eGFR slopes, only values which were obtained before starting tolvaptan were used to obtain an insight into natural disease progression characteristics. Using these values was sufficient to calculate eGFR slopes for 992 patients in a therapy-naïve state. Of these, 958 passed the criteria of being below 5 and above -15 ml/min/1.73 m²/yr. These analyses showed a faster eGFR decline in male patients (−3.2 ml/min/1.73 m²/yr compared to −2.8 ml/min/1.73 m²/yr in female patients, Fig. 2D). Annual kidney function loss increased with increasing MIC as expected (1A -2.0 ml/min/1.73 m²/yr, 1B -2.2 ml/min/1.73 m²/yr, 1C-3.0 ml/min/1.73 m²/yr, 1D -3.7 ml/min/1.73 m²/yr, 1E -4.3 ml/min/1.73 m²/yr, Fig. 2E). MIC 2 patients showed a mean eGFR slope compatible with slow disease progression (−1.8 ml/min/1.73 m²/yr; Fig. 2E). Besides showing aggregated data, MEDA can be used to provide study centers with individual patient-level data after secure dual authentication login (Fig. S9A). This is especially useful for visualizing individual eGFR slopes, e.g., to assess timing towards future onset of kidney failure, in a way ensuring data security (Fig. S9B, C, also see section on “individual patient” dashboard below).

### Usage of and adherence to tolvaptan in the real-life setting

**Fig. 3:**
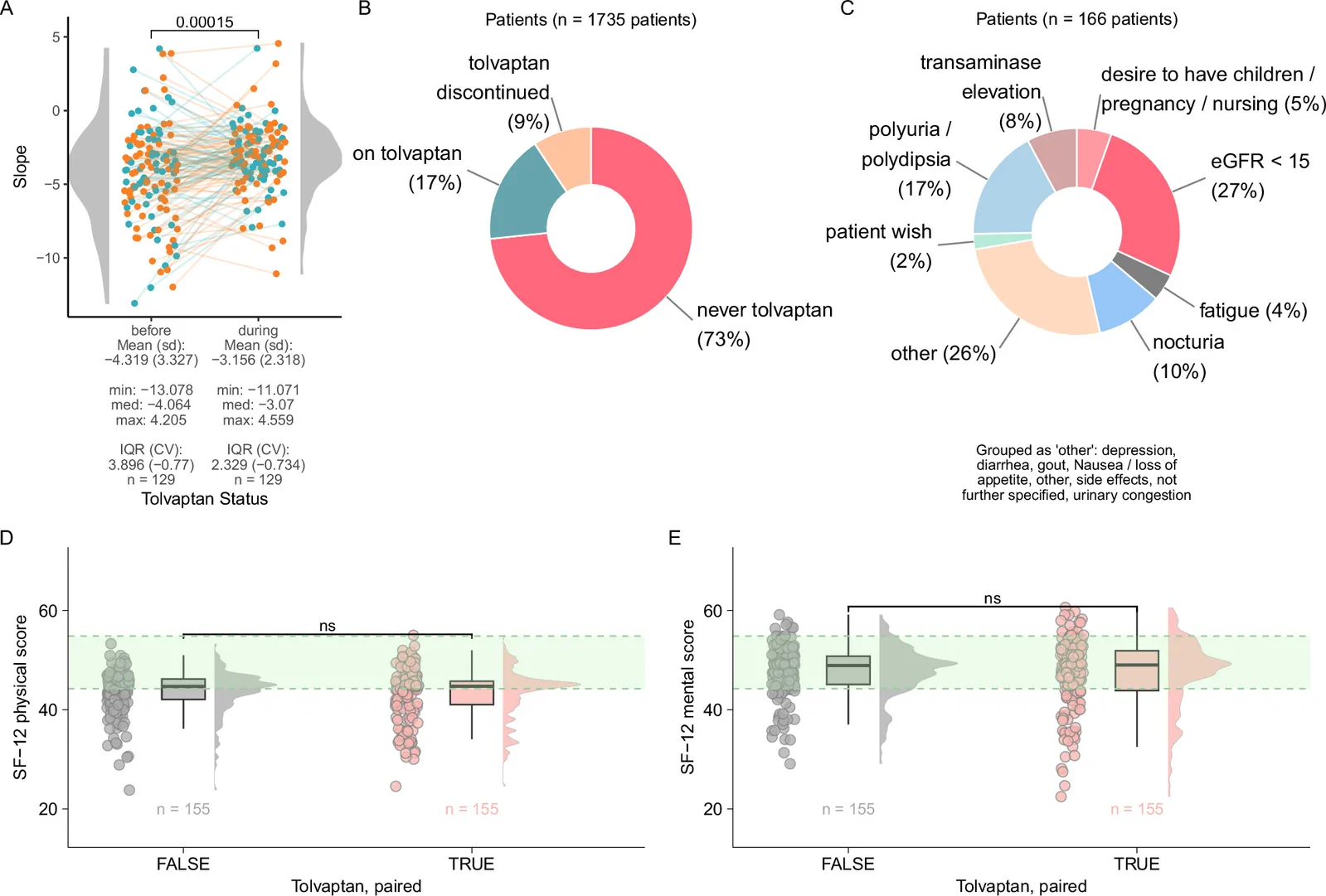
Dashboard “Tolvaptan” (tolvaptan usage and tolerability), effect of tolvaptan on eGFR slope (dashboard “Kidney function decline”) and quality of life (dashboard “Quality of life”). **A** Longitudinal paired analysis of eGFR slopes before and on tolvaptan (using all eGFR values available). **B** Proportion of patients who were on tolvaptan at any visit during follow-up across the entire cohort. **C** Reasons for discontinuation of tolvaptan. **D** Quality of life assessed using the SF-12 physical score in a paired analysis of patients before and on tolvaptan. Green-shaded area depicts the distribution in the normal population. **E** Quality of life assessed using the SF-12 mental score in a paired analysis of patients before and on tolvaptan. Green-shaded area depicts the distribution in the normal population. **A**: “Kidney Function Decline” Dashboard; **B-C**: “Tolvaptan” Dashboard; **D-E**: “Quality of Life” Dashboard.

The majority of patients were not on tolvaptan and did not start tolvaptan during follow-up. Approximately 26% of patients took tolvaptan at any point during data collection and - out of these - 37% had stopped tolvaptan during follow-up (Fig. 3B). In these 166 patients, polyuria/nocturia was the most common reason for discontinuation (27.7%) followed by reaching an eGFR of <15 ml/min/1.73 m^2^ (26.5%; Fig. 3C). Transaminase elevation was reported as the cause for stopping tolvaptan in 9% of all patients that discontinued the treatment (Fig. 3C).

### Effectiveness of tolvaptan on kidney function decline and quality of life in the real-life setting

We performed a longitudinal paired analysis of the impact of tolvaptan on eGFR decline in all patients for whom sufficient creatinine data was available over a period of at least one year before taking tolvaptan and while being on treatment. Within our dashboard tool we chose to utilize a simplified comparison based on directly comparing calculated eGFR slopes using a t-test. This approach showed a highly significant amelioration of kidney function loss by approximately 27% (*p*-value 0.00015; Fig. 3A). When assessing the slope differences in a sex specific manner both reached statistical significance, with men showing greater response than women (men: *p*-value 0.0012, women: *p*-value 0.035 Fig. S10A, B). To improve on this statistical analysis, we also applied a linear mixed-effects model to all eGFR values of patients who at some point received tolvaptan without imposing the minimum eGFR value availability criterion specified in the methods (≥3 values over ≥1 year). This model showed that the estimated rate of decline improved from −3.62 ml/min/1.73 m²/yr before treatment to −3.01 ml/min/1.73 m²/yr during treatment, an absolute attenuation of 0.61 ml/min/1.73 m²/yr (95% CI 0.07 to 1.16; *p* = 0.028), corresponding to a relative reduction in the rate of decline of 16.9% (95% CI 3.3–30.6%). Independently of this slope effect, initiation was associated with a significant acute reduction in eGFR level of approximately 2.5 ml/min/1.73 m² (95% CI 1.1–3.9; *p* < 0.001), consistent with the expected hemodynamic effect of tolvaptan. There was no significant effect modification by sex (treatment-by-sex interaction on slope, *p* = 0.18). The apparent difference between the sexes can therefore be related to differences in subgroup sample size and statistical power rather than a genuine difference in treatment effect (Table 2). Quality of life was assessed using the SF-12 tool. While the physical health score was comparable, the mental health score appeared to be better on tolvaptan in cross-sectional analyses comparing all questionnaires completed on tolvaptan with those completed off tolvaptan (Fig. S11A, B). The same holds true when comparing the first questionnaire completed by patients who never took tolvaptan to the first questionnaire of patients filled after starting tolvaptan (Fig. S11C, D). However, these differences were no longer evident in a longitudinal paired analysis for all patients that had filled at least one questionnaire before and on tolvaptan. This analysis showed no tolvaptan-dependent differences in either the mental or the physical health score (Fig. 3D, E). Consequently, tolvaptan treatment does not substantially impair overall SF-12 physical or mental health scores despite its well-recognized burden of aquaretic side effects.

**Table 2:** Results of the effect of tolvaptan on patients in the AD(H)PKD cohort using a linear mixed effect model

Parameter	Estimate	95% CI (*p*-value)
Pre-treatment eGFR slope	- 3.62 ml/min/1.73 m²/yr	-
On-treatment eGFR slope	- 3.01 ml/min/1.73 m²/yr	-
Change in slope on tolvaptan	0.61 ml/min/1.73 m²/yr	0.07 to 1.16 (*p* = 0.028)
Relative reduction in decline rate	16.9%	3.3–30.6%
Acute change in eGFR level at initiation	- 2.5 ml/min/1.73 m²	−3.9 to −1.1 (*p* < 0.001)
Treatment x sex interaction (slope)	Not significant	*p* = 0.18

### Individual patient dashboard enabling a patient-specific overview of ADPKD-related key characteristics

**Fig. 4:**
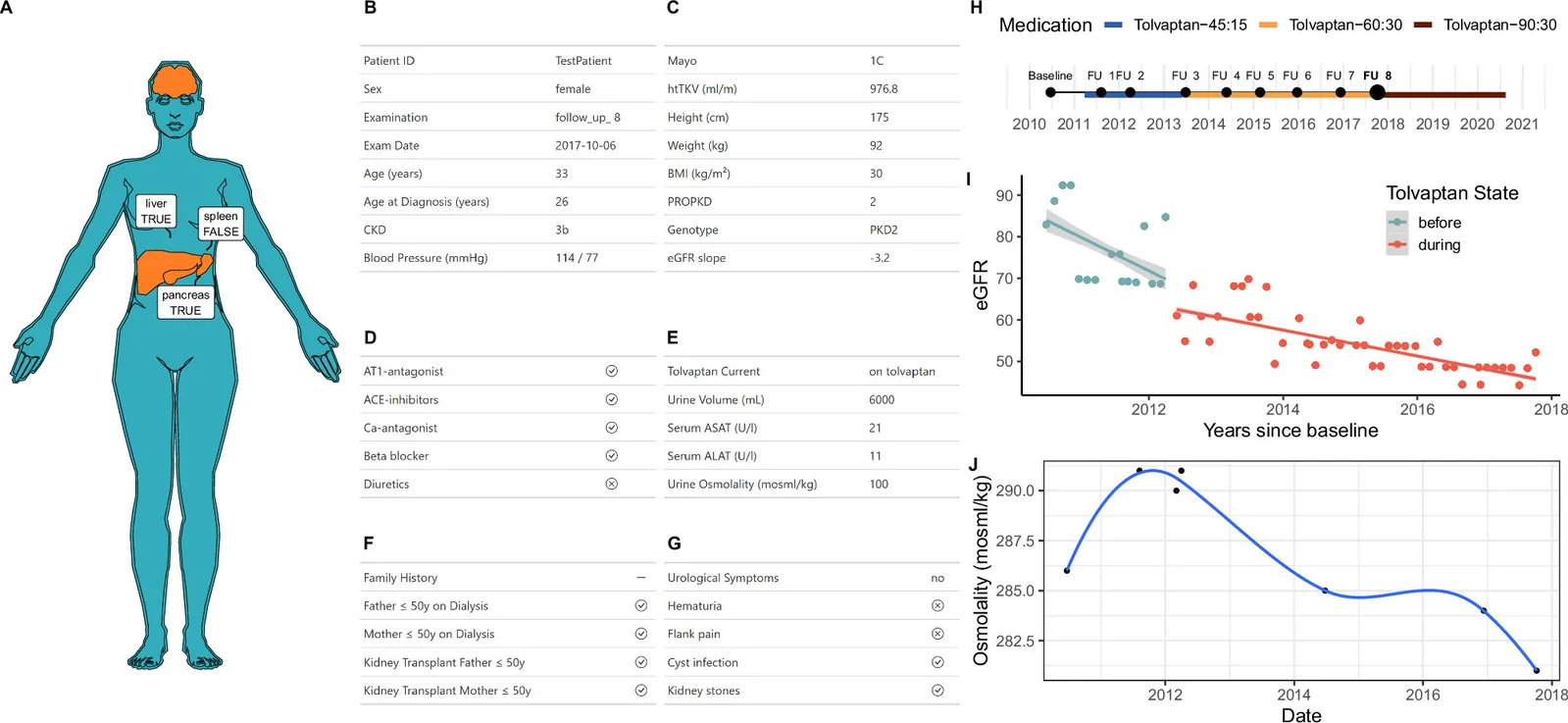
Individual Patient dashboard. **A** Extrarenal cysts as known at the selected visit. **B** Patient characteristics at the selected visit (shown in bold) and age at diagnosis. **C** Predictors of disease progression. **D** Co-medication classes at the selected visit. (✔ = yes, x = no). **E** Key visit specific lab values. **F** Family history (+ = positive, - = negative, ✔ = yes, x = no). **G** Urological manifestations (+ = positive, - = negative, ✔ = yes, x = no). **H** Visit schedule over time indicating tolvaptan treatment phases and dosage, visits can be selected by clicking upon them. **I** eGFR slope indicating phases with and without tolvaptan. **J** Laboratory values can be selected freely and the selected value is then visualized over time (example ALAT).

To allow for insight into the effect of disease progression and the impact of tolvaptan in individual patients, we established an additional dashboard. This dashboard is not freely available to respect data protection on a single-patient level. Access is provided to participating centers through a dual authentication-based login (Fig. S9A). Once logged in, treating clinician-scientists can select individual patients from their cohort to get a full overview of key characteristics important to patient care in ADPKD summarized on one page. This includes an overview of all visits at the center visualizing phases with tolvaptan treatment and respective dosage (Fig. 4H). In addition, key characteristics are shown including sex, age, age at diagnosis, blood pressure and CKD stage (Fig. 4B). Extrarenal cysts are visualized on a body map (Fig. 4A) and parameters predicting outcome are summarized including MIC, BMI, PROPKD score, genotype and eGFR slope (based on values without tolvaptan, Fig. 4C). Furthermore, the dashboard includes family history (Fig. 4F) and urological symptoms (Fig. 4G). Importantly, eGFR slope is visualized and shown separately for phases with and without tolvaptan allowing to assess response and to extrapolate the time until kidney failure (Fig. 4I). The user can select individual lab values from the full set which are then visualized in the dashboard over time (Fig. 4J). Key concomitant medication is shown by classes (Fig. 4D) providing a quick insight into whether relevant treatments have been implemented. Since, besides the scientific analyses possible with MEDA-PKD, supporting both clinical trials and clinical care is a key future goal of the tool, we obtained a first insight into the impact MEDA-PKD could have in these settings. Using the MEDA-PKD filters for a set of key inclusion (eGFR, MIC) and exclusion characteristics (tolvaptan use) of the currently active STOP-PKD trial (https://stop-pkd.de/) showed that 595 patients in the current cohort would be eligible in principle at this point in time. While further checks on the resulting list of patients are admittedly necessary and refinement for the actual details of criteria in individual trials will be required as a second step, this analysis takes 10–15 s, can directly be followed by automated extraction of patient IDs facilitating pre-screening and can serve as a valid argument for feasibility analyses. To provide an exploratory assessment of the usability of the system, seven clinicians, experienced with the care for patients with ADPKD, were asked to assess the tool using the 10-item System Usability Scale (SUS)^19^ and a set of ADPKD-specific questions. The resulting overall SUS score was 90.4, which is generally considered an excellent usability rating (Fig. S12A), in line with their assessment of the use of MEDA-PKD specifically for the setting of patient counseling in ADPKD (Fig. S12B). The same assessors continued with a test of time requirements. When comparing to data readiness in the established patient information system, which had previously been filled with all required data used in routine care, to obtain information required to assess the indication for tolvaptan, MEDA-PKD resulted in highly significant time savings (mean 72.4%, group means 634.5 to 172.3 s, *p* < 0.001, Fig. S13).

### Integrating omics data with clinical data to identify biomarkers for relevant outcome parameters

**Fig. 5:**
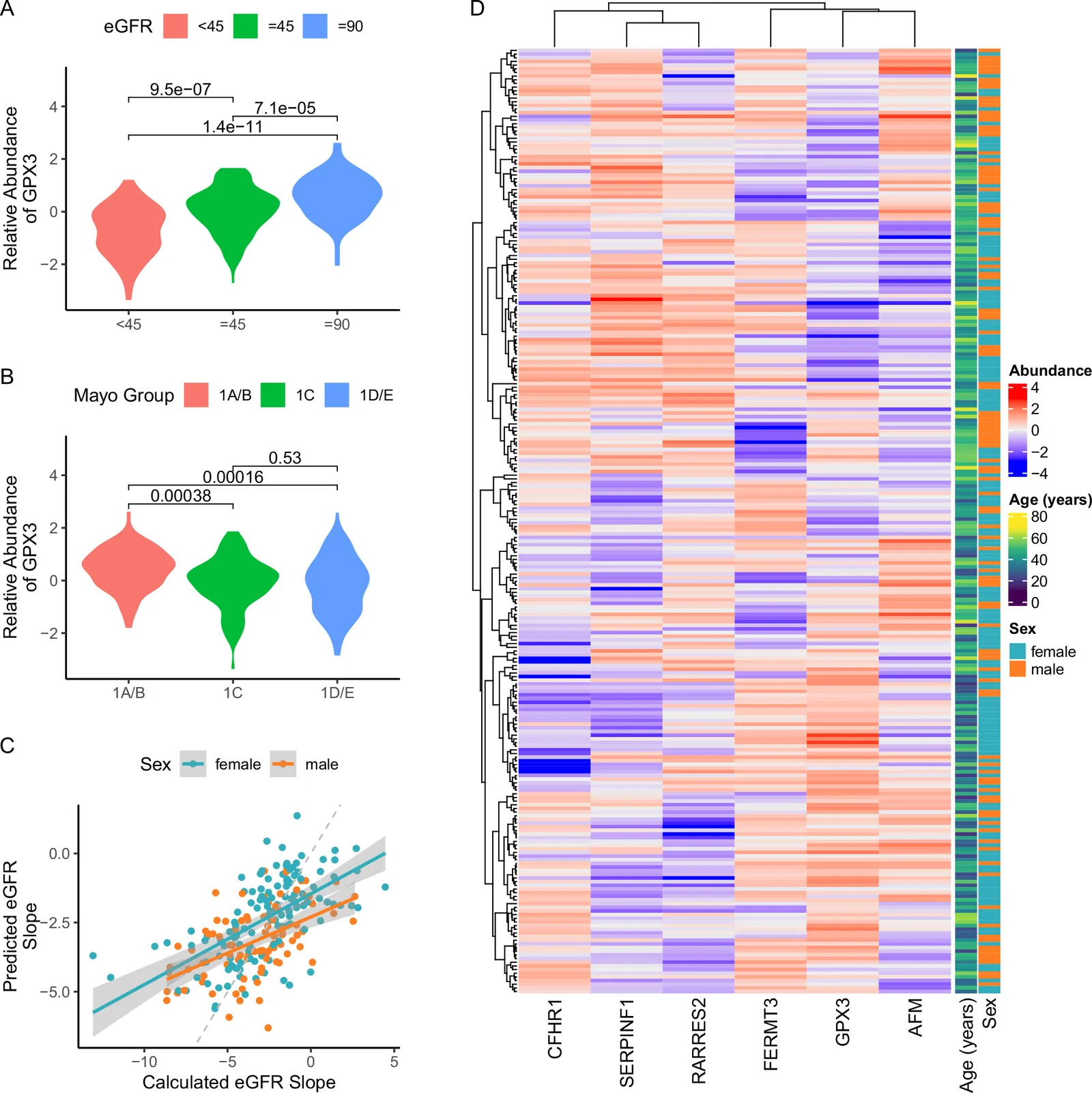
Integrating omics data with clinical data to identify biomarkers for relevant outcome parameters (OMICS dashboard). **A** Relative protein abundance of proteins in the Aydogan-Balaban et al. serum proteome dataset depicted by eGFR classes (GPX3 shown as an example). **B** Relative protein abundance of proteins in the Aydogan-Balaban et al. serum proteome dataset depicted by Mayo Imaging Classes (GPX3 shown as an example). **C** Plot showing prediction accuracy of the six-protein model based on real-time updates of eGFR slopes. **D** Heatmap of protein abundance in conjunction with clinical parameters, both can be freely chosen by the user (showing the proteins from the Aydogan-Balaban et al. six-protein model and age/sex as examples).

We have recently published a serum proteomics dataset on a substantial proportion of the AD(H)PKD cohort identifying a set of proteins predicting eGFR slope^20^. MEDA-PKD now allows the user to query this dataset to examine the association of proteins of choice with markers of disease severity in ADPKD linking omics data to clinical parameters in a user-defined fashion. The user can define any protein identified in the dataset and visualize its abundance across different levels of eGFR or different MIC (shown for GPX3 in an exemplary manner in Fig. 5A). Besides, any protein of choice can be added to a heatmap in which clinical parameters can be added freely by the user (Fig. 5B). Most importantly, while the original publication provides the static model performance of a six-protein model (including SERPINF1, GPX3, AFM, FERMT3, CFHR1 and RARRES2) MEDA-PKD now links this model to the actual continuously updated slopes from the cohort and visualizes calculated vs. predicted eGFR slope (Fig. 5C). To not limit biomarker identification to research omics data but also allow the user to explore the relation of classical clinical laboratory values to markers of disease severity, MEDA-PKD features a “Lab Value” dashboard allowing the user to access 214 laboratory parameters. Graphs showing the impact of tolvaptan on urine volume and urine osmolality as well as transaminases are always shown in the dashboard as standard parameters (Fig. S14A–D). Besides, the user can select any of the 214 variables to be visualized by sex, MIC, CKD stage and tolvaptan status (Fig. S14E–G).

## Discussion

The MEDA-PKD platform makes an important step toward near-real-time precision nephrology and illustrates how routinely collected clinical and molecular data can be transformed into a continuously learning ecosystem. By linking longitudinal registry data with hospital information systems, imaging, and omics resources through an automated, harmonized infrastructure, MEDA-PKD transcends the limitations of traditional static registries. Rather than providing single snapshots of aggregated data, it enables dynamic exploration of disease trajectories and treatment effects as data accrue, closing the long-standing gap between data generation, analysis, and clinical translation^21–23^.

From a clinical perspective, the results generated within MEDA-PKD confirm established patterns of ADPKD progression, such as sex- and TKV-based differences in eGFR decline^24,25^, while providing the granularity to detect therapy effects under real-world conditions. The observed real-world 27% reduction in eGFR loss on therapy with tolvaptan is in line with the effect size observed in the pivotal randomized clinical trials (RCTs)^12,13^ and underscores the platform’s capacity to evaluate therapeutic interventions in near-real-time. When eGFR slopes were obtained through an automated longitudinal update rather than retrospective reanalysis (using a linear mixed effects model), effectiveness is maintained, but comes with a smaller effect size 16.9% (CI: 3.3–30.6%). The observed difference in CI in the mixed-effects model indicates that not all patients benefit from tolvaptan intake. It is important to note, that this approach used all data available for all patients starting tolvaptan while the t-test based approach imposed a minimum requirement of 3 eGFR values over a period of ≥1 year. Nonetheless, it will now be of major interest to identify factors influencing effectiveness in individual patients including actual adherence. Importantly, integration of patient-reported outcomes and lifestyle variables extends the interpretability of these findings beyond renal endpoints, offering a more comprehensive view of disease burden and treatment benefit. This capacity to continuously evaluate both biological and patient-centered outcomes is essential to accelerate precision medicine in chronic kidney disease and other complex disorders^26,27^. In this regard, the integration of total liver volume (TLV)-associated progression groups in patients affected by polycystic liver disease would be of interest^28^. Furthermore, different layers of outcome prediction in ADPKD could be integrated as recently performed for PROPKD score and MIC^29^.

Technically, MEDA introduces several key innovations that differentiate it from existing registry or data warehouse approaches. The platform’s modular architecture, combining a dockerized PostgreSQL backend with an R/Shiny-based analytics interface, ensures scalability, reproducibility, and interoperability. Automated data ingestion from multiple sources - including clinical information systems, research databases, and omics data in a unique manner - minimizes manual curation while maximizing data availability and reduces error-prone data transfer. Continuous integration via Jenkins and version control through GitHub guarantee that every analytical and visualization process is transparent, traceable, and reproducible. These design principles enable MEDA to function as a “living” data resource in which new information directly enriches existing analyses without additional manual intervention^18^.

Compared to established infrastructures such as the ERA and ERKReg kidney disease registries^30,31^, or large-scale frameworks like CKDGen^32^ and the UK Biobank^33^, MEDA differs in both granularity and immediacy. Most existing registries operate on static, periodic data exports and require centralized re-analysis to generate new insights. While these efforts have provided invaluable large-scale population data, their architectures are not designed for near-real-time interaction or automated integration of multi-omic datasets at this point. In contrast, MEDA enables clinicians and researchers to dynamically explore longitudinal data, stratify patient subgroups, and link molecular signals to clinical phenotypes without additional programming effort. This immediacy creates an unprecedented opportunity for rapid hypothesis generation, validation of clinical observations, pre-screening for clinical trials and identification of therapeutic response patterns under routine care conditions.

This framework will be of benefit to many stakeholders in the field and can serve as a basis for hypothesis generation and validation to scientists, for trial design to pharmaceutical industry and for patient information and involvement through lay language versions. In this regard, the inclusion of patient-reported outcome measures (PROMs) such as the SF12 questionnaire is of special interest. The dual authentication protected internal version allows individual centers to visualize single-patient level data, e.g., to inform treatment decisions or as a useful addition to patient counseling. Near-real-time visualization of treatment responses makes real-life cohort data accessible for an assessment of treatment effectiveness, e.g., valuable to policymaking in the healthcare reimbursement sector, and the impact of previously unstudied therapeutic combinations or subgroup analyses. Moreover, this approach provides insight into real-life tolerability of side effects, adherence and causes of treatment cessation^11^.

Beyond the specific context of ADPKD, the MEDA framework provides a scalable blueprint for translational data infrastructures in other diseases. Its data model and dashboard modules are disease-agnostic and can be adapted to different clinical contexts by modifying variable dictionaries and analytics templates. For example, the same technical backbone could support cohorts focusing, e.g., on diabetes mellitus-associated CKD, but also heart failure, cystic fibrosis, or cancer. Extension to existing cohort platforms such as ERKReg and integration with data infrastructures such as the German National Research Data Infrastructure (NFDI4Health)^34^ or the German Portal for Medical Research Data (FDPG)^35^ is readily achievable via standardized APIs and data harmonization pipelines. By supporting federated analyses while preserving local data ownership, MEDA addresses key regulatory and ethical barriers that have historically limited multicenter data integration^36,37^.

The technical backbone of MEDA-PKD is intentionally generic and was originally validated in a disease-agnostic implementation by Schmidt et al.^18^, to which we refer readers for the full technical specification, including dependency versions and example deployment scripts. The minimum infrastructure required to deploy a MEDA-PKD–like instance comprises a Linux server (or virtual machine) capable of running Docker; a PostgreSQL instance for the harmonized backend with off-site daily backups; an R/Shiny server (or equivalent visualization layer); a continuous-integration tool (we use Jenkins) to schedule the Python ingest scripts; and network access to the local hospital information system through standardized APIs (FHIR or vendor-specific export) and, where applicable, to a registry platform such as clinicalsurveys.net or REDCap. As all components are open source, the platform can be reproduced without proprietary licenses. Generalizability is principally constrained by the availability of structured data in the local hospital information system, interoperability with the relevant APIs (which typically requires local IT engagement), and governance arrangements for the use of routine clinical data in research, which vary substantially between jurisdictions. We regard these as actionable barriers rather than fundamental limitations and address them through a roadmap for federated extension to additional centers via standardized variable mapping. Specifically, our planned roadmap comprises three steps: (i) publication of a standardized variable dictionary and mapping templates to harmonize data from external centers; (ii) onboarding of additional German tertiary and non-tertiary centers via the existing API-based ingestion pipeline; and (iii) federated analyses across sites that preserve local data ownership, building on integration with infrastructures such as ERKReg, NFDI4Health, and others.

In the broader landscape of digital health research, MEDA contributes a unique combination of interoperability, automation, and interpretability. Whereas most hospital data warehouses or FAIR-compliant research databases focus on data availability^38^, MEDA emphasizes accessibility and usability turning data into directly interpretable, hypothesis-generating visualizations. The open-source analytical scripts and modular container design also ensure sustainability: the platform can be maintained, extended, and reused across institutions without dependence on proprietary software ecosystems. In this way, MEDA bridges the traditional divide between clinical registries, translational research platforms, and clinical decision support tools^39,40^ and makes an important contribution to FAIR data sharing strategies. Besides, external centers can submit their data after variable mapping in a harmonized format to build cohort-specific subtools and provide access to individual patient dashboard views.

Several limitations and future perspectives warrant consideration. First, while the platform provides continuous automated data ingestion and quality control, completeness and accuracy remain dependent on underlying source systems. Potential differences in documentation practices, varying levels of structured data capture across participating centers and residual missingness may lead to information bias. Ongoing harmonization efforts and increasing adoption of structured electronic health records will further enhance data fidelity. Second, current implementations of the omics integration module focus on proteomics^20^; expansion to genomics, metabolomics, and imaging-derived radiomics is underway^41^. The proteomics subcohort currently comprises only a fraction of the full MEDA-PKD cohort, as described in Aydogan-Balaban, Arjune et al.^20^. While this subcohort is generally a random sample regarding clinical characteristics, residual enrichment is still possible, considering that MIC 2 patients were excluded and the fact that inclusion of patients with available TKV data and sufficient follow-up was a selection criterion to enter proteome measurement and model generation. MEDA-PKD is based on a prospective cohort which was initially conceived to follow patients considered for tolvaptan therapy. This design may have preferentially captured individuals with more aggressive disease phenotypes, kidney function compatible with treatment recommendations and access to detailed radiological characterization, thereby introducing a degree of selection bias. In addition, because participating sites are predominantly German tertiary referral centers, the cohort lacks broad ancestral, ethnic, and socioeconomic representation. Differences in genetic determinants of disease, healthcare accessibility, and management practices across populations limit the direct transferability of these findings to underrepresented patient groups. Besides, user assessment was not conducted in a blinded manner due to lack of feasibility of such an approach and is limited in user number at this point. It should thus be considered exploratory. Nonetheless, the exploratory usability analysis with an overall SUS score > 90 in combination with the ADPKD-specific feedback and the time-savings indicate very good usability. The results regarding the impact of tolvaptan on eGFR slope do not reflect randomized clinical trial standards and have to be interpreted as real-world observational data. However, such findings on effectiveness are especially important in the setting of a drug like tolvaptan which due to polyuria may be limited in adherence. For the ADPKD use-case addressed by MEDA-PKD it will now be important to continue development by continuously adding new data layers currently being established. This will include further omics datasets from various biosample types, radiomics analyses of MRI images and deeper analyses of psychosocial distress.

Finally, sustained engagement by clinicians and researchers will be essential to realize the full potential of MEDA as a community resource. Practical barriers that may slow adoption in clinical life are integrating new data workflows into clinical environments and reducing documentation burden.

Long-term support of MEDA-PKD relies on the ongoing availability of resources for infrastructure management, software development and updates, as well as personnel skilled in clinical data management and computational sciences. The open-source framework and modular system architecture provide substantial flexibility and reduce dependence on proprietary technologies. MEDA-PKD is jointly operated by the Department of Internal Medicine II (University Hospital Cologne), the Department of Nephrology at University Hospital Düsseldorf, the Medical Data Integration Center (MeDIC) Cologne, and the Core-IT Facility of the Medical Faculty Cologne, whose shared provision of hardware and personnel secures maintenance and operating costs for at least the next ten years. As the virtual-machine–based infrastructure can be freely scaled and relocated within existing university resources, and as several stakeholders have already approached us, we expect collaborations with public funders and industry to support sustainable further development. However, durable implementation will require funding models that are not restricted to short-term project financing or industry contributions. In conclusion, despite these challenges, MEDA-PKD exemplifies how digital infrastructure can transform medical cohorts into dynamic, self-updating ecosystems for precision medicine. Its application spans clinical care, trial enrichment, biomarker discovery, and post-marketing drug surveillance. MEDA-PKD provides a scalable, interoperable, and transparent platform that not only advances the understanding and management of ADPKD but also offers a generalizable template for other diseases. By coupling real-world clinical data with automated analytics and translational integration, MEDA moves beyond static registry concepts toward a continuously learning health system – a cornerstone for next-generation biomedical research^42^.

## Methods

### Study design and cohort

This analysis is based on data from the German AD(H)PKD Tolvaptan Registry (NCT02497521, DRKS00008910), an ongoing multicenter, longitudinal observational study designed to collect real-world data from adults (≥18 years) with ADPKD. The registry systematically documents clinical, laboratory, imaging, and patient-reported data in individuals undergoing evaluation or treatment with tolvaptan in routine care or study follow-up.

### Ethical approval statement

All participants provided written informed consent prior to inclusion. The study protocol was approved by the Ethics Committee of the University of Cologne and all participating institutions. The study is conducted in accordance with the Declaration of Helsinki and the Good Clinical Practice (GCP) guidelines of the International Council for Harmonization (ICH). The study is publicly registered at ClinicalTrials.gov, NCT02497521 and the German Clinical Trials Register, DRKS00008910. Informed consent was obtained from all participants and the study had been approved (ethical approval number 15-323) by the Ethics Committees of all participating centers (i.e., of the Universities of Cologne, Würzburg, Lübeck, Leipzig, Tübingen, and Medizinische Hochschule Hannover as well as the Ärztekammer Sachsen, Schleswig-Holstein and Baden-Württemberg).

### Inclusion criteria

Eligible participants were adults (≥18 years) with clinically or genetically confirmed ADPKD who were followed at participating nephrology centers and evaluated for initiation or continuation of tolvaptan therapy.

### Data collection and structure

Clinical data are collected prospectively and updated at least annually, with additional visits scheduled for patients initiating tolvaptan. Data capture follows a standardized electronic case report form using the secure web-based platform clinicalsurveys.net^43^.

The dataset includes (but is not limited to):

- Demographics: age, sex, family history
- Genotype data
- Kidney function: serum creatinine, eGFR (CKD-EPI)
- Imaging parameters: total kidney volume (TKV) measured by MRI or CT and categorized by MIC.
- Medication: tolvaptan, antihypertensive medication, statins and opioids.
- Extrarenal manifestations: hepatic cysts, cardiovascular findings
- Lifestyle and patient-reported outcomes: smoking, alcohol consumption, and standardized quality-of-life questionnaires (SF-12)^44^.
- Laboratory values: >200 parameters covering electrolytes, liver function, metabolic, and inflammatory markers.

All data undergo automated quality control, plausibility checks, and harmonization across sites to ensure comparability and completeness.

### MEDA database and integration of diverse data sources

On the backend MEDA runs on a dockerized PostgreSQL database. Here all the data is stored, and backups generated daily as well as stored securely off-site. To ingest data into the database, several Python scripts run at various intervals during the day. First, data is extracted from clinicalsurveys.net through their standalone application programming interfaces (APIs)^18^. These data are restructured and injected into the database. Next, routine clinical data is extracted from the medical information system of the hospital and imported. Finally, to enable additional user entry in a familiar style to clinicians we employ a NocoDB instance linked to the PostgreSQL database. This provides a web-based table interface and enables data entry of information that is not captured by the remaining systems. In addition, the R/Shiny interface, introduced in the next section, also has the ability to import more static data, such as omics data or other associated datasets.

### Integration of omics data

To enable deep phenotyping and translational research, MEDA-PKD incorporates molecular data alongside clinical information. The current version includes a serum proteomics dataset from the Cologne AD(H)PKD subcohort^20^. Within the Aydogan-Balaban et al. publication, this data has identified a six-protein model which is able to predict eGFR slope and is implemented within MEDA-PKD. As more data becomes available the protein abundance data are continuously linked to clinical outcomes such as eGFR slope, MIC class, and treatment exposure. In addition, users can select proteins of interest and visualize associations dynamically across patient subgroups (e.g., sex, CKD stage, tolvaptan use).

### MEDA visualization

The MEDA-PKD platform was designed to provide an interactive, near-real-time environment for the visualization and analysis of longitudinal ADPKD registry data. Visualization modules were developed using R/Shiny, integrating the ggplot2 and plotly libraries to enable dynamic generation of time-series plots, stratified comparisons, and correlation analyses across multiple clinical domains. Data from the PostgreSQL backend are accessed through standardized application programming interfaces (APIs), allowing near-real-time rendering of graphics and ensuring consistency and reproducibility of all outputs.

The platform enables stratified visualization of clinical trajectories according to sex, age group, CKD stage, MIC, tolvaptan treatment status, or genotype. Within the kidney function dashboard, longitudinal eGFR curves and estimated slopes are visualized for individual patients as well as aggregated subgroups, providing an intuitive overview of disease progression. Imaging parameters, including total kidney volume (TKV), can be examined by MIC class and related directly to renal function decline. Medication use, adherence, and discontinuation are visualized over time to facilitate assessment of treatment effects in real-world settings. Extrarenal manifestations such as hepatic cysts or cardiovascular anomalies are represented as frequency distributions, while patient-reported outcomes, including lifestyle factors and standardized quality-of-life measures, are displayed in longitudinal and cross-sectional formats.

A dedicated laboratory explorer allows for interactive visualization of 214 routinely measured parameters through customizable comparative boxplots, supporting the identification of biochemical patterns across treatment and disease subgroups. Advanced analytics functions enable users to perform subgroup analyses and regression modeling directly. For example, the eGFR Slope Explorer calculates and visualizes the annual decline in kidney function for selected patient groups, with options to overlay treatment status or biomarker levels. The omics integration module connects molecular data, such as proteomics, with clinical trajectories and automatically updates associations as new data are added to the registry.

All visualization outputs can be exported as static or interactive figures containing metadata to ensure transparency and traceability. Analytical scripts used within the platform are version-controlled via GitHub and automatically deployed through Jenkins within a continuous integration framework, guaranteeing reproducibility of all visual and analytical processes (https://github.com/nephro-adpkd/meda). By transforming static cohort data into an interactive analytical ecosystem, MEDA-PKD empowers clinicians and researchers to generate hypotheses, evaluate therapeutic responses, and explore biomarker-disease relationships in near-real-time, thereby advancing precision medicine in ADPKD. Further information for the user on how to use the platform is also provided in the screencast video accompanying the publication at https://doi.org/10.18716/cecad/adpkd_1080p.

### Real-life user assessment

7 clinicians experienced in the care for patients with ADPKD and not involved in the development of MEDA-PKD were asked to assess the tool using structured questionnaires, specifically the System Usability Scale (SUS) and ADPKD-specific questions. The SUS is an established industry-standard 10-item questionnaire designed to obtain a measure of how people perceive the usability of computer systems^19^. For reporting, the SUS scores are converted to a range of 0 to 100 with 100 indicating maximum usability (described in more detail in the corresponding publication). The ADPKD-specific questions were designed specifically by us for this purpose (see Fig. S12). The same users went on to measure the time required for obtaining information on the key items needed for patient counseling on tolvaptan (see Fig. S13A). Access to these data items in the established clinical information system (ORBIS, Dedalus), which had previously been filled with all data available on these patients using a structured approach in the setting of an ADPKD expert center, served as a comparator.

### Statistical analyses

Statistical analyses in MEDA are governed by the underlying data distribution. Group comparisons used chi-square or Fisher’s exact tests for categorical variables (e.g., sex differences in tolvaptan use) and one-way ANOVA or Kruskal-Wallis for continuous variables (e.g., eGFR slopes by MIC class), with post-hoc Tukey or Dunn’s tests as appropriate. Two-group continuous comparisons used unpaired t-tests (normal) or Mann-Whitney U (non-normal). Values that are not normally distributed are log transformed prior to their analysis. For the predictive model, a linear regression model for eGFR slope from Aydogan-Balaban et. al.^20^ is integrated directly into MEDA. eGFR slopes are calculated by a simple robust linear model predicting eGFR as a function of date. At least three values over a one-year period are required for a slope to be calculated. The resulting coefficient is then multiplied by 365.25 to represent an annual eGFR slope for an individual. Within the tool we applied a simplified approach to measure the impact of tolvaptan by comparing off and on tolvaptan calculated slopes through a simple t-test and report the resulting *p*-value. To improve the estimation of the effect on the trajectory of kidney function, we further fit a linear mixed-effects model (R package nlme) to all available serum creatinine-based eGFR measurements from patients who eventually received tolvaptan. Time was expressed in days relative to the date of tolvaptan initiation. The fixed-effects structure used a change-point (“hinge”) parameterization anchored at the initiation date, comprising: (i) a continuous time term capturing the pre-treatment slope; (ii) a term equal to time elapsed since initiation (zero beforehand), whose coefficient estimates the change in slope attributable to tolvaptan; and (iii) a binary treatment indicator switching from 0 to 1 at initiation, whose coefficient estimates the acute change in eGFR level at the start of treatment. The model was adjusted for age, sex and Mayo Imaging Class. We specified random intercepts and random pre- and on-treatment slopes per patient, with a continuous-time first-order autoregressive correlation structure for the within-patient residuals. Models were fitted by REML. Effect modification by sex was tested by adding the corresponding interaction terms; as these were non-significant, they were removed from the final model. The relative reduction in the rate of decline and its 95% confidence interval were derived from the model coefficients using the delta method. Statistical testing of the time required for data collection in the user test was performed using a paired Student’s *t* test.

Fitted lines generated within the laboratory values over time, are based on a loess smoothed curve with standard parameters. Apart from the proteomics data, for which imputation is described in the original publication^20^, no data is imputed in the context of MEDA-PKD. For clinical data, missing data are removed prior to analysis and all sample sizes are reported.

## Supplementary information

Suppl_Figures_04092026

## Data Availability

The datasets generated and/or analyzed during the current study are not publicly available in order to ensure data protection of study participants and minimize the risk of re-identification but are available from the corresponding authors on reasonable request and after signing a data transfer agreement. Alternatively, analyses performed by the coordinating centers and provision of aggregated data can be requested through the corresponding authors.

## Code availability

The generic MEDA core (data transformation and database layer) is openly available under the MIT licence on GitHub (https://github.com/nephro-adpkd/meda) and archived on Zenodo [45]. The MEDA-PKD-specific ingestion scripts and R/Shiny dashboards are available from the corresponding authors on reasonable request. All docker images used are standard, publically available, images provided and all dashboards apart from the individual patient level data can be freely accessed.

## References

[1] Ong, A. C. M., Devuyst, O., Knebelmann, B. & Walz, G. Autosomal dominant polycystic kidney disease: the changing face of clinical management. *Lancet* **385**, 1993–2002 (2015). 10.1016/S0140-6736(15)60907-2 26090645

[2] Müller, R.-U. & Benzing, T. Management of autosomal-dominant polycystic kidney disease—state-of-the-art. *Clin. Kidney J.* **11**, i2–i13 (2018). 10.1093/ckj/sfy103 PMC6295602 30581561

[3] Spithoven, E. M. et al. Renal replacement therapy for autosomal dominant polycystic kidney disease (ADPKD) in Europe: prevalence and survival-an analysis of data from the ERA-EDTA Registry. *Nephrol. Dial. Transplant.* **29**, iv15–iv25 (2014). 10.1093/ndt/gfu017 PMC7611099 25165182

[4] Miskulin, D. C. et al. Health-related quality of life in patients with autosomal dominant polycystic kidney disease and CKD stages 1-4: a cross-sectional study. *Am. J. Kidney Dis.* **63**, 214–226 (2014). 10.1053/j.ajkd.2013.08.017 PMC4075014 24183837

[5] Müller, R.-U. et al. KDIGO 2025 ADPKD guideline: a commentary on diagnosis and management of hepatopancreatic manifestations by the ERA Working Group Genes & Kidney. *Nephrol Dial Transpl.* 10.1093/ndt/gfaf159. PMC12836103 40810467

[6] Arjune, S., Todorova, P., Bartram, M. P., Grundmann, F. & Müller, R.-U. Liver manifestations in autosomal dominant polycystic kidney disease (ADPKD) and their impact on quality of life. *Clin. Kidney J.* sfae363 10.1093/ckj/sfae363.(2024). PMC11852259 40008356

[7] Arjune, S. et al. Cardiac manifestations in patients with autosomal polycystic kidney disease (ADPKD) - a single-center study. *Kidney360* 10.34067/KID.0002942022 (2022). PMC10103268 36821607

[8] Cornec-Le Gall, E., Torres, V. E. & Harris, P. C. Genetic complexity of autosomal dominant polycystic kidney and liver diseases. *J. Am. Soc. Nephrol.* 10.1681/ASN.2017050483 (2017). PMC5748917 29038287

[9] Lanktree, M. B. et al. Intrafamilial variability of ADPKD. *Kidney Int Rep.* **4**, 995–1003 (2019). 10.1016/j.ekir.2019.04.018 PMC6611955 31317121

[10] Müller, R.-U. et al. An update on the use of tolvaptan for autosomal dominant polycystic kidney disease: consensus statement on behalf of the ERA Working Group on Inherited Kidney Disorders, the European Rare Kidney Disease Reference Network and Polycystic Kidney Disease International. *Nephrol. Dial. Transplant.* **37**, 825–839 (2022). 10.1093/ndt/gfab312 PMC9035348 35134221

[11] Todorova, P. et al. Interaction between determinants governing urine volume in patients with ADPKD on Tolvaptan and its impact on quality of life. *Kidney Int. Rep.* 10.1016/j.ekir.2023.05.011 (2023). PMC10403673 37547529

[12] Torres, V. E. et al. Tolvaptan in patients with autosomal dominant polycystic kidney disease. *N. Engl. J. Med.* **367**, 2407–2418 (2012). 10.1056/NEJMoa1205511 PMC3760207 23121377

[13] Torres, V. E. et al. Tolvaptan in later-stage autosomal dominant polycystic kidney disease. *N. Engl. J. Med.* **377**, 1930–1942 (2017). 10.1056/NEJMoa1710030 29105594

[14] Coates, A. et al. The use and role of digital technology in learning health systems: a scoping review. *Int. J. Med. Inform.* **178**, 105196 (2023). 10.1016/j.ijmedinf.2023.105196 37619395

[15] Inau, E. T., Sack, J., Waltemath, D. & Zeleke, A. A. Initiatives, concepts, and implementation practices of FAIR (Findable, Accessible, Interoperable, and Reusable) data principles in health data stewardship practice: protocol for a scoping review. *JMIR Res. Protoc.* **10**, e22505 (2021). 10.2196/22505 PMC7886612 33528373

[16] Foley, T. & Horwitz, L. I. Learning Health Systems. *Elem. Improving Qual. Saf. Healthc.* 10.1017/9781009325912 (2025).

[17] Pannunzio, V., Kleinsmann, M., Snelders, D. & Raijmakers, J. From digital health to learning health systems: four approaches to using data for digital health design. *Health Syst.* **12**, 481–494 (2023). 10.1080/20476965.2023.2284712 PMC10791080 38235300

[18] Schmidt, J. et al. Bridging health registry data acquisition and real-time data analytics. *Front. Med*. **11**, 1430676 (2024). 10.3389/fmed.2024.1430676 PMC11716499 39790555

[19] Brooke, J. SUS: a retrospective. *J. Usability Stud.* **8**, 29–40 (2013).

[20] Aydogan Balaban, H. Ö et al. Developing serum proteomics based prediction models of disease progression in ADPKD. *Nat. Commun.* **16**, 6646 (2025). 10.1038/s41467-025-61887-8 PMC12274525 40681500

[21] Gehrmann, J., Herczog, E., Decker, S. & Beyan, O. What prevents us from reusing medical real-world data in research. *Sci. Data* **10**, 459 (2023). 10.1038/s41597-023-02361-2 PMC10345145 37443164

[22] Schneeweiss, S. Real-world evidence of treatment effects: the useful and the misleading. *Clin. Pharm. Ther.* **106**, 43–44 (2019). 10.1002/cpt.1405 30942896

[23] Steel, P. A. D., Wardi, G., Harrington, R. A. & Longhurst, C. A. Learning health system strategies in the AI era. *npj Health Syst.* **2**, 21 (2025). 10.1038/s44401-025-00029-0 PMC13354208 42527455

[24] Cornec-Le Gall, E. et al. The PROPKD score: a new algorithm to predict renal survival in autosomal dominant polycystic kidney disease. *J. Am. Soc. Nephrol.* **27**, 942–951 (2016). 10.1681/ASN.2015010016 PMC4769200 26150605

[25] Irazabal, M. V. et al. Imaging classification of autosomal dominant polycystic kidney disease: a simple model for selecting patients for clinical trials. *J. Am. Soc. Nephrol.* **26**, 160–172 (2015). 10.1681/ASN.2013101138 PMC4279733 24904092

[26] Ashley, E. A. The precision medicine initiative: a new national effort. *JAMA* **313**, 2119–2120 (2015). 10.1001/jama.2015.3595 25928209

[27] El-Achkar, T. M. et al. Precision medicine in nephrology: an integrative framework of multidimensional data in the kidney precision medicine project. *Am. J. Kidney Dis.* **83**, 402–410 (2024). 10.1053/j.ajkd.2023.08.015 PMC10922684 37839688

[28] PLD-Progression Grouper. https://halbritter-lab.github.io/pld-progression-grouper/).

[29] Wolff, C. A. et al. Integrated use of autosomal dominant polycystic kidney disease prediction tools for risk prognostication. *Clin. J. Am. Soc. Nephrol.* **20**, 397–409 (2025). 10.2215/CJN.0000000625 PMC11906014 39705090

[30] Bassanese, G. et al. The European rare kidney disease registry (ERKReg): objectives, design and initial results. *Orphanet J. Rare Dis.* **16**, 251 (2021). 10.1186/s13023-021-01872-8 PMC8173879 34078418

[31] Boerstra, B. A. et al. The ERA Registry Annual Report 2021: a summary. *Clin. Kidney J.* **17**, sfad281 (2024). 10.1093/ckj/sfad281 PMC11024806 38638342

[32] Wuttke, M. et al. A catalog of genetic loci associated with kidney function from analyses of a million individuals. *Nat. Genet* **51**, 957–972 (2019). 10.1038/s41588-019-0407-x PMC6698888 31152163

[33] Bycroft, C. et al. The UK Biobank resource with deep phenotyping and genomic data. *Nature* **562**, 203–209 (2018). 10.1038/s41586-018-0579-z PMC6786975 30305743

[34] Abaza, H. et al. Toward a domain-overarching metadata schema for making health research studies FAIR (Findable, Accessible, Interoperable, and Reusable): development of the NFDI4Health metadata schema. *JMIR Med. Inf.* **13**, e63906 (2025). 10.2196/63906 PMC12138291 40397930

[35] FDPG - Forschungsdatenportal für Gesundheit. https://forschen-fuer-gesundheit.de/en/.

[36] Rieke, N. et al. The future of digital health with federated learning. *npj Digit. Med.* **3**, 119 (2020). 10.1038/s41746-020-00323-1 PMC7490367 33015372

[37] Weiner, E. B., Dankwa-Mullan, I., Nelson, W. A. & Hassanpour, S. Ethical challenges and evolving strategies in the integration of artificial intelligence into clinical practice. *PLOS Digit Health* **4**, e0000810 (2025). 10.1371/journal.pdig.0000810 PMC11977975 40198594

[38] Pigeot, I., Intemann, T., Kollhorst, B., Sax, U. & Ahrens, W. [FAIRification of real world data for health research]. *Pravent Gesundh* 1–8 10.1007/s11553-022-00973-x. (2022). PMC9516507 40479265

[39] Evans, R. S. Electronic health records: then, now, and in the future. *Yearb Med. Inform.* **25**, S48–S61 (2016). 10.15265/IYS-2016-s006 PMC5171496 27199197

[40] Hripcsak, G. & Albers, D. J. Next-generation phenotyping of electronic health records. *J. Am. Med. Inf. Assoc.* **20**, 117–121 (2013). 10.1136/amiajnl-2012-001145 PMC3555337 22955496

[41] Eddy, S., Mariani, L. H. & Kretzler, M. Integrated multi-omics approaches to improve classification of chronic kidney disease. *Nat. Rev. Nephrol.* **16**, 657–668 (2020). 10.1038/s41581-020-0286-5 32424281

[42] Friedman, C. et al. Toward a science of learning systems: a research agenda for the high-functioning Learning Health System. *J. Am. Med. Inf. Assoc.* **22**, 43–50 (2015). 10.1136/amiajnl-2014-002977 PMC4433378 25342177

[43] ClinicalSurveys.net. https://www.clinicalsurveys.net/uc/main/2890/loft/front.php?module=loft&controller=login.

[44] Johnson, J. A. & Coons, S. J. Comparison of the EQ-5D and SF-12 in an adult US sample. *Qual. Life Res.* **7**, 155–166 (1998). 10.1023/a:1008809610703 9523497

