# Supplementary material for: MEDA-PKD: a dynamic, real-time analytics platform for precision care in autosomal dominant polycystic kidney disease
Source: NPJ Digit Med. 2026 Oct 20;9:791. doi: 10.1038/s41746-026-03267-0 (PMC13683356; doi:10.1038/s41746-026-03267-0)
Supplement: Supplementary file 1 [file 41746_2026_3267_MOESM1_ESM.pdf]

MEDA-PKD: A Dynamic, Real-Time Analytics Platform for Precision Care in Autosomal Dominant Polycystic Kidney Disease

Supplemental Figures

Figure S1: Outline of the technical infrastructure and data sources underlying MEDA-PKD

Figure S2: Filters available in the online tool

Figure S3: Cohort statistics

Figure S4: Supplements to baseline characteristics and extrarenal manifestations

Figure S5: Dashboard Family History

Figure S6: Dashboard “Medication“ providing information on usage of the most important drug classes in ADPKD

Figure S7: Dashboard “Lifestyle“ providing information on smoking, coffee and alcohol consumption.

Figure S8: Total kidney volume, arterial hypertension and urological complications

Figure S9: Individual patient-level eGFR trajectories including treatment effects

Figure S10: Treatment effect of tolvaptan by sex

Figure S11: The impact of tolvaptan on quality of life as assessed by the SF-12 questionnaire

Figure S12: Insights into real-life usability of MEDA-PKD

Figure S13: Quantification of time-savings enabled through MEDA-PKD by real-life testers

Figure S14: Dashboard „Lab Values“

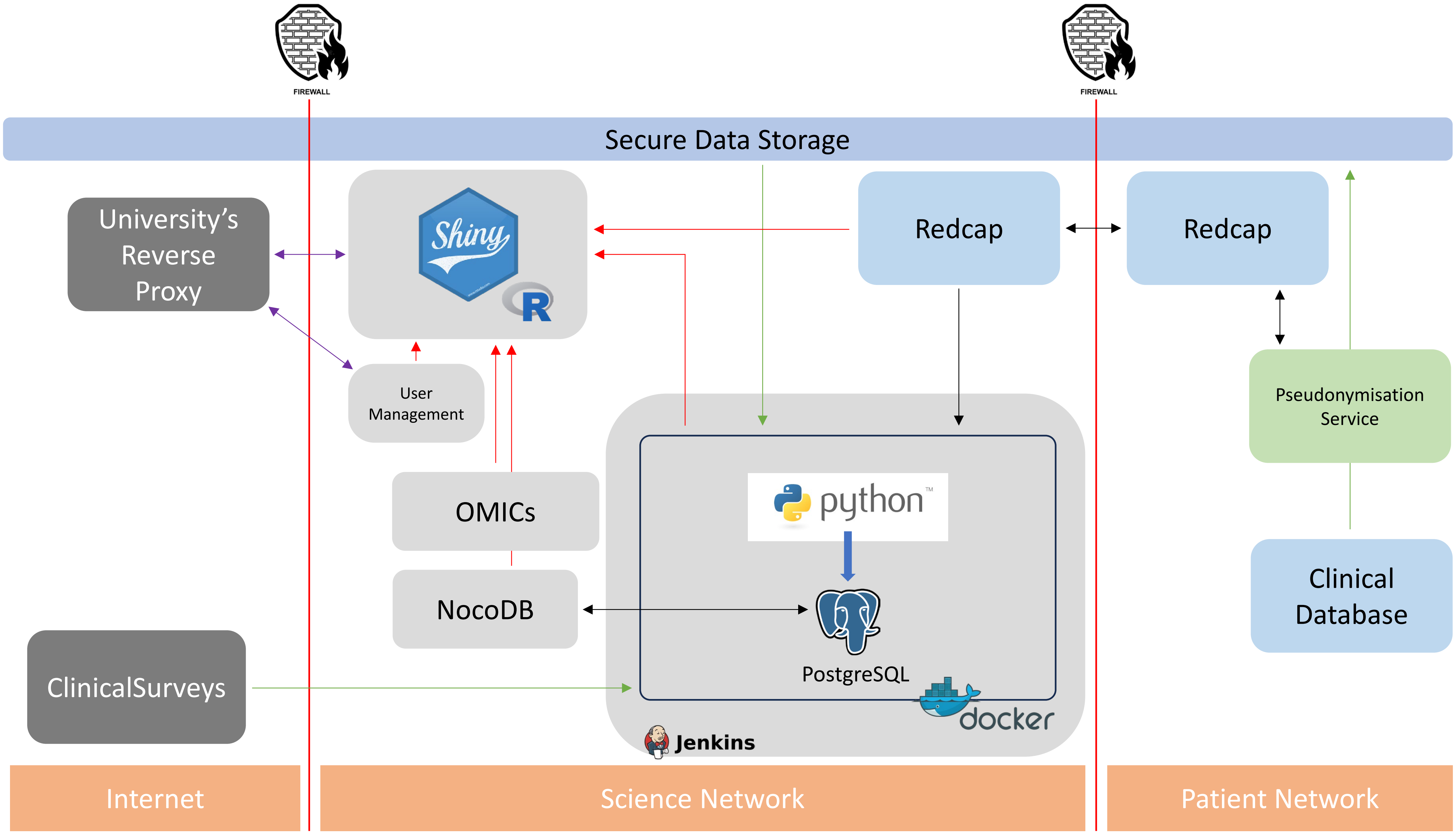

Figure S1: Outline of the technical infrastructure and data sources underlying MEDA-PKD

MEDA unifies various internal and external datasources into a normalized PostgreSQL database as a basis for Shiny-driven visualization and analysis. Laboratory values, basic demographics, ICD-coded diagnoses and visit dates are pulled on a daily basis through standardized APIs directly from the clinical information system. Additionally, various other data sources can be integrated. Currently, this includes clinical information on co-morbidities, pharmacological treatment, family history, dietary habits and questionnaires such as quality of life tools which are entered manually into eCRF tools (e.g. Clinical Surveys or NocoDB). In general, fully structured databases can be directly integrated into the PostgreSQL database (e.g. proteome data, NocoDB) while unstructured datasources are first transformed into a normalized database format employing an established Python-based algorithm. The entire process of data integration into the PostgreSQL database as well as data visualization and statistical analysis is fully automated through Jenkins. Additional datasources can be integrated into this workflow at any time. Green arrows represent daily update cycles, while red arrows represent an on demand cycle checking every 10 min whether new data has been made available. Black arrows represent an on demand cycle without new data check. Purple arrows define a simple https connection between the reverse proxy and our webservices.

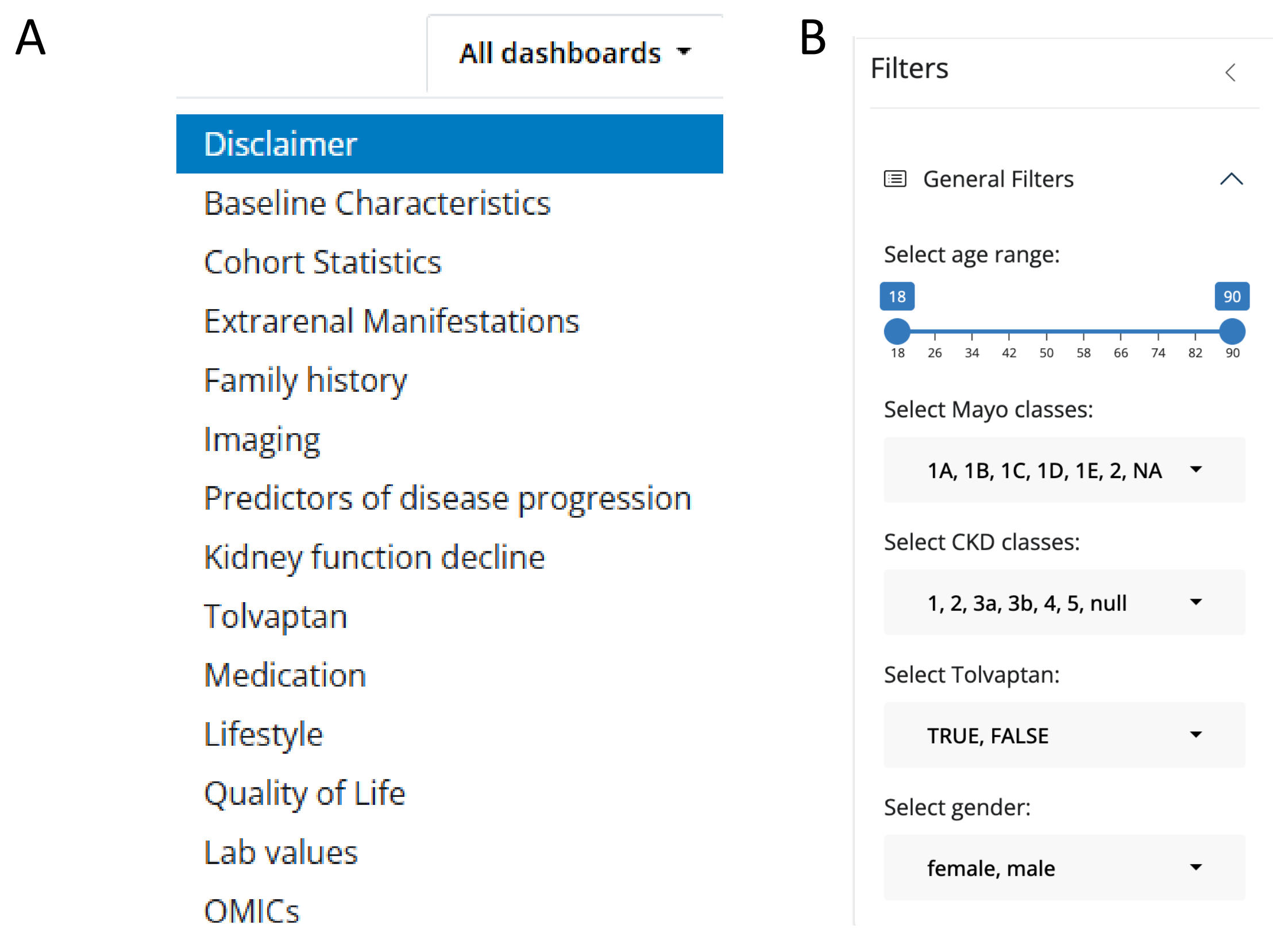

Figure S2: Filters available in the online tool

A MEDA-PKD provides several dashboards containing specific sets of information. All of the visualizations are updated in a real-time manner upon entry of new data into the database or automatic extraction of data (e.g. lab values) from the clinical information system. These tabs include General patient characteristics,cohort statistics family history, medication, extrarenal manifestations, imaging, predictors of disease progression (e.g. genotype, Mayo Imaging Class), annual eGFR decline, information on tolvaptan use, general lifestyle characteristics, quality of life and lab values.Besides, visualizations of serum proteome data linked to key clinical outcome parameters are accessible.

B To ensure a dynamic and versatile platform for the user, all dashboards can be filtered based on key clinical characteristics such as age, Mayo Imaging Class, CKD stage, Tolvaptan usage and sex.

A

Cohort Statistics

	Overall (N=1676)
**Age**	
Mean (SD)	43.9 (12.8)
Median [Min, Max]	44.0 [18.0, 83.0]
**htTKV**	
Mean (SD)	1020 (847)
Median [Min, Max]	778 [132, 9870]
Missing	158 (9.4%)
**Weight**	
Mean (SD)	82.7 (20.0)
Median [Min, Max]	80.0 [40.0, 189]
Missing	25 (1.5%)
**Height**	
Mean (SD)	175 (11.9)
Median [Min, Max]	175 [61.0, 209]
Missing	23 (1.4%)
**eGFR**	
Mean (SD)	68.9 (31.9)
Median [Min, Max]	66.6 [6.51, 143]
Missing	22 (1.3%)

	Overall (N=1676)
**Sex**	
female	864 (51.6%)
male	812 (48.4%)
**Tolvaptan**	
Yes	358 (21.4%)
No	1318 (78.6%)
**Family History**	
Negative	322 (19.2%)
Positive	1353 (80.7%)
Unknown	1 (0.1%)
**Mayo**	
1A	82 (4.9%)
1B	339 (20.2%)
1C	481 (28.7%)
1D	362 (21.6%)
1E	197 (11.8%)
2	68 (4.1%)
NA	147 (8.8%)

B

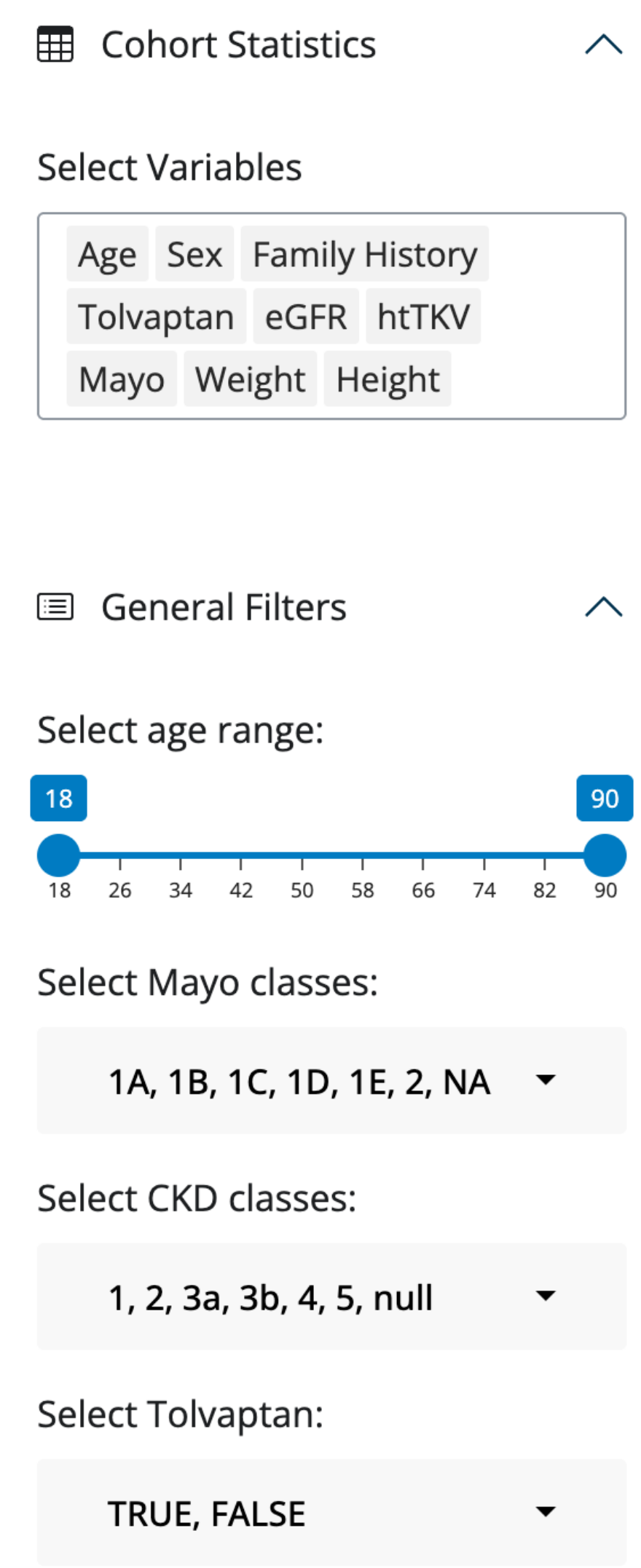

Figure S3: Cohort statistics

A Cohort statistics table as shown in the online MEDA-PKD tool when using default settings.
B This table can be adapted by filtering the cohort or adding / removing individual characteristics to be shown.

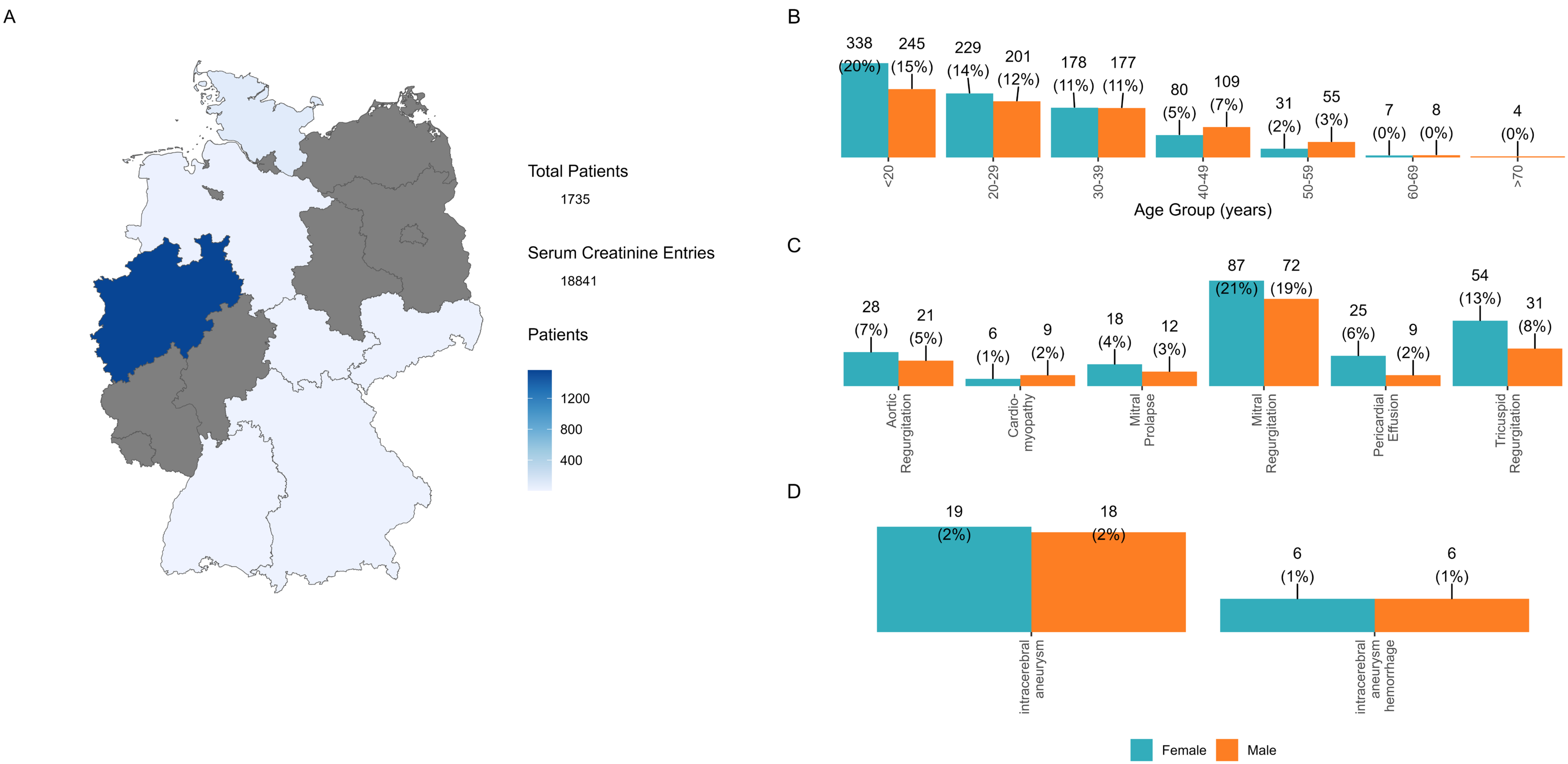

Figure S4: Supplements to baseline characteristics and extrarenal manifestations

A Distribution of patient recruitment across Germany. In total, data on 1735 patients was collected as of March 3, 2026 across 7 federal states including 18,841 serum creatinine values.

B Age at first diagnosis of ADPKD by sex.

C Echocardiographic findings in female and male patients

D Intracranial aneurysms and hemorrhage in male and female patients

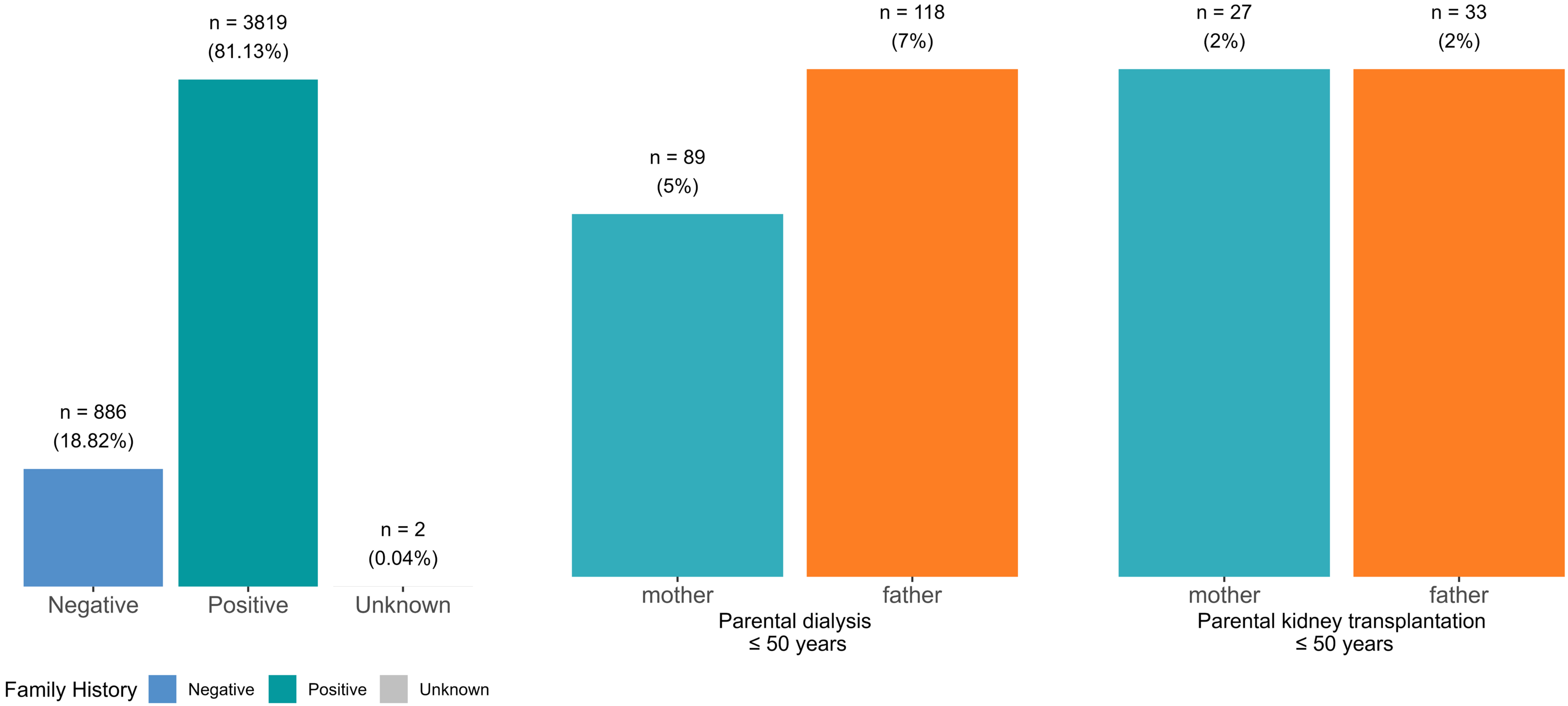

Figure S5: Dashboard Family History

A Family history of cohort participants.

B Fraction of patients reporting early onset of dialysis in an affected parent.

C Fraction of patients reporting early kidney transplantation in an affected parent.

Entry	Completeness
Patient ID	100.0%
Examination	99.3%
Sex	100.0%
Age at Diagnosis	95.9%
Age	100.0%
Race	100.0%
Tolvaptan Current	96.7%
Tolvaptan Ever	100.0%
htTKV	90.2%
Family History	100.0%
Exam Date	100.0%
TKV	90.2%
Weight	97.7%
Height	97.4%
Arterial Hypertension	99.7%
Intracerebral Aneurysms	44.7%
Pancreatic Cysts	68.6%
Pancreatic Neoplasia	66.3%
Urological Symptoms	98.8%
Systolic BP	96.3%
Diastolic BP	96.3%
Kidney stones	100.0%
Cyst infection	100.0%
Flank pain	100.0%
Hematuria	100.0%
Kidney Transplant Father ≤ 50y	100.0%
Kidney Transplant Mother ≤ 50y	100.0%
Father ≤ 50y on Dialysis	100.0%
Mother ≤ 50y on Dialysis	100.0%
Mayo	100.0%
Mutation	54.7%
eGFR slope	57.2%
Creatinine	97.5%
eGFR	97.5%
CKD	100.0%
PROPKD	25.1%
Genotype	43.2%

Table S1: Completeness of key Variables used within the Dashboard as of March 3rd 2026

Completeness of key variables used within the Dasbhoard show that apart from Intracerebral Aneurysms (45%), Pancreatic Cysts (68%), Pancreatic Neoplasia (66%), Mutational status (55%), eGFR slope (71%), PROPKD Score (25%), and Genotype (43%), variables have a completeness of > 90%.

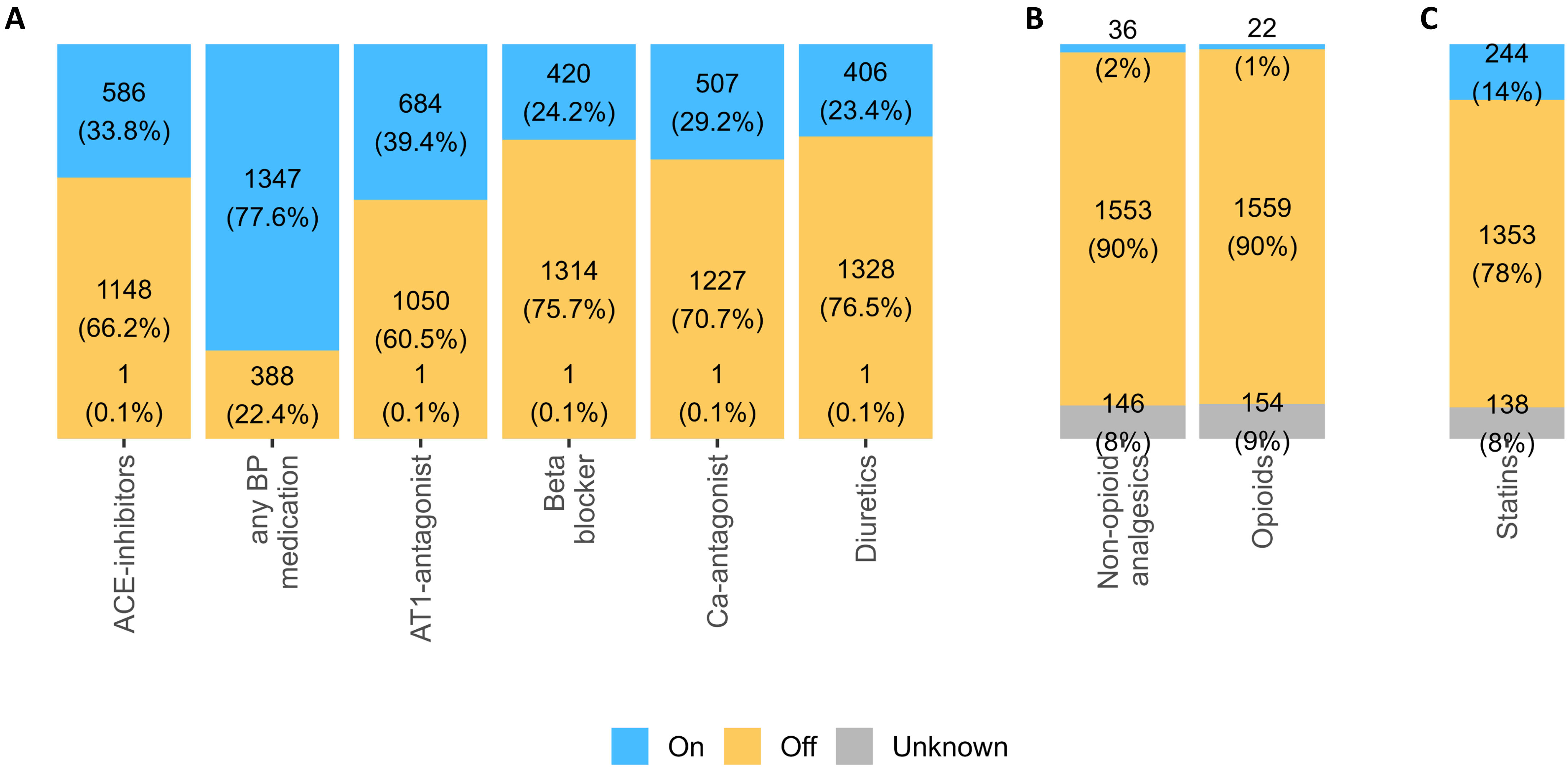

Figure S6: Dashboard "Medication" providing information on usage of the most important drug classes in ADPKD

A Antihypertensive medication classes.

B Pain medication.

C Statin treatment.

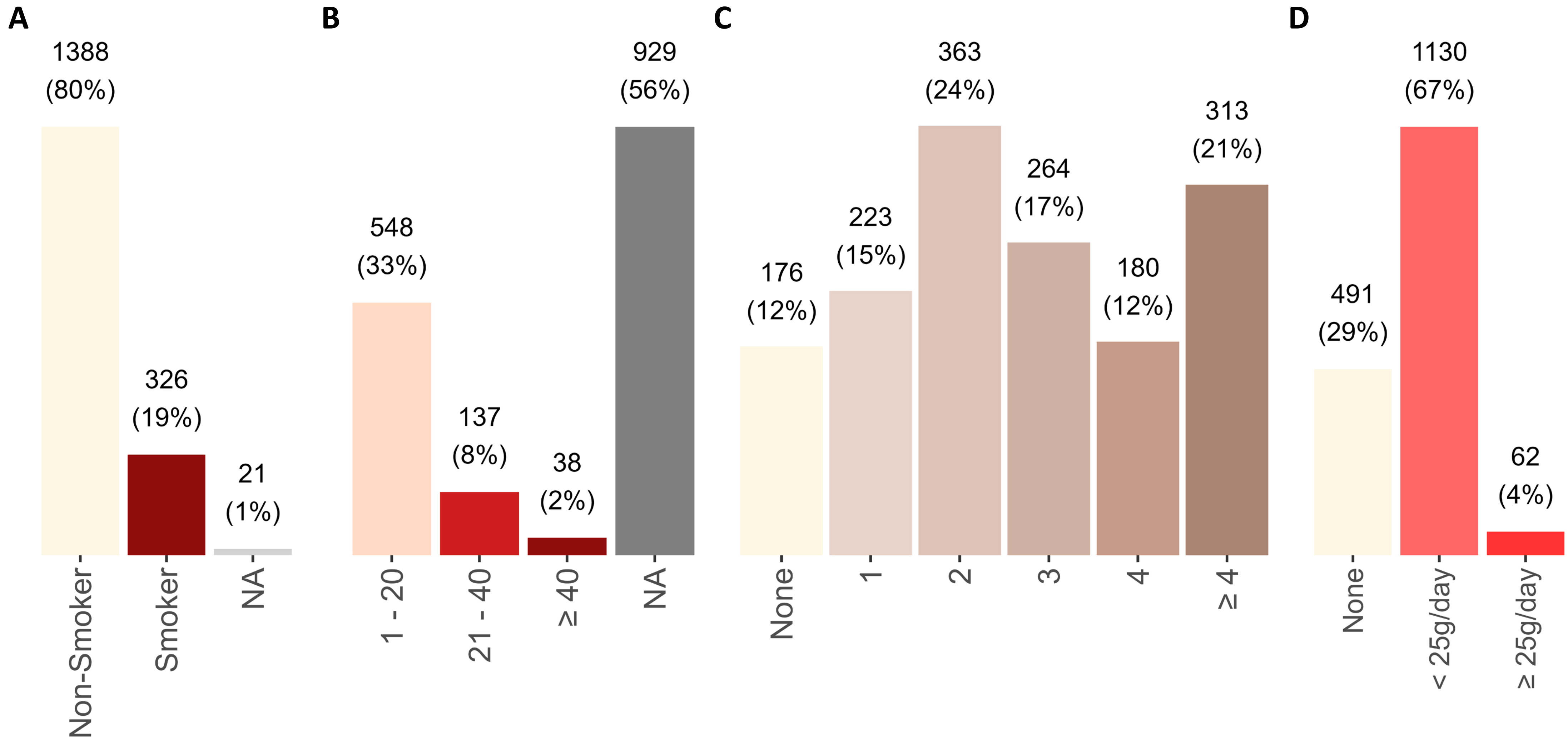

Figure S7: Dashboard “Lifestyle“ providing information on smoking, coffee and alcohol consumption

A Proportion of active smokers in the cohort.

B Pack years. NA are non-smokers: participants who never smoked, neither in the past nor at inclusion.

C Coffee consumption in cups per day.

D Alcohol consumption in grams per day.

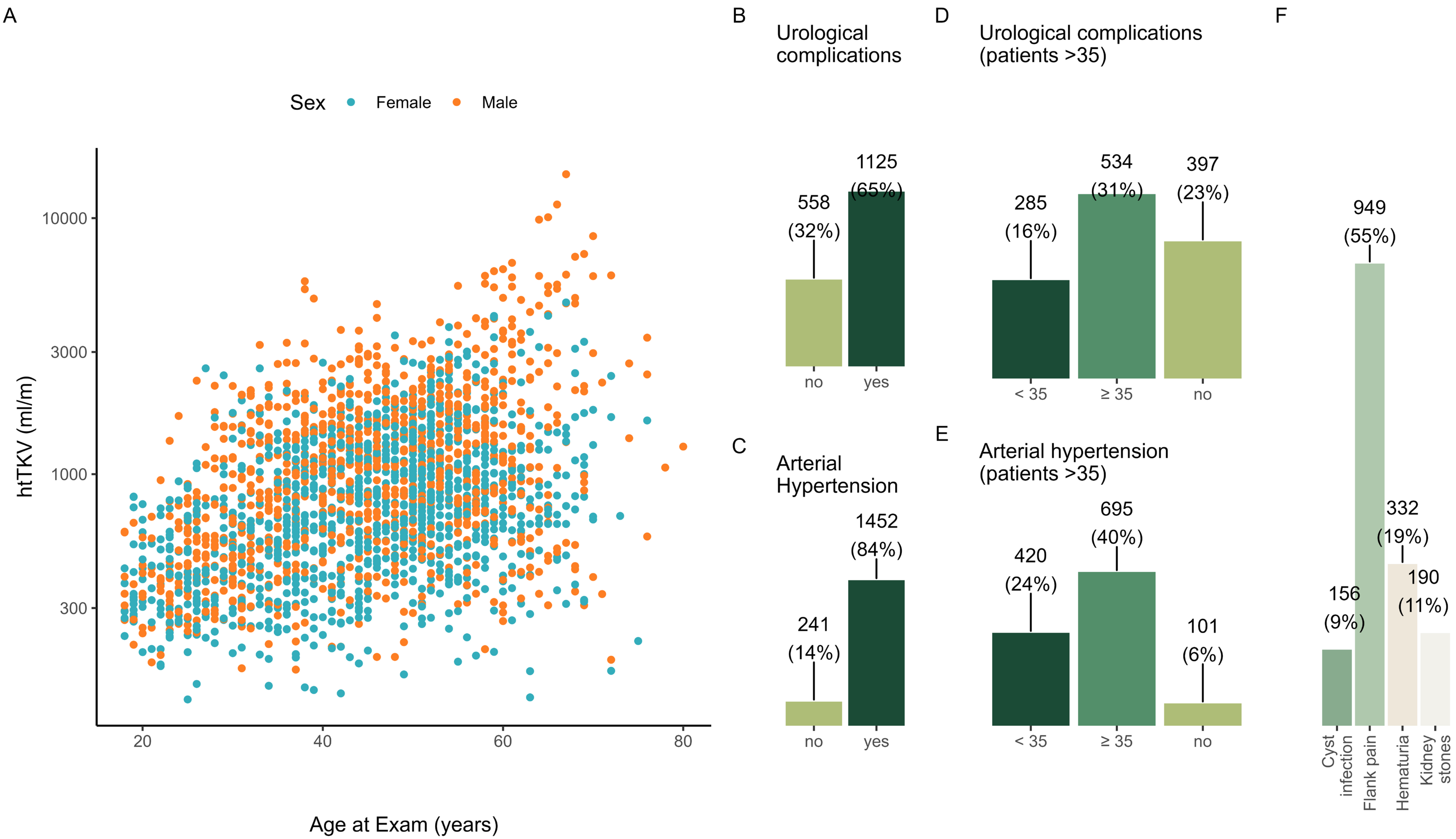

Figure S8: Total kidney volume, arterial hypertension and urological complications

A Height-adjusted total kidney volume (htTKV) by sex and age (Dashboard „Imaging“)

B Urological complications in the entire cohort (hemorrhagic events involving gross hematuria or cyst hemorrhages, cyst infections, or flank pain related to cysts; Dashboard „Predictors of Disease Progression).

C Arterial hypertension in the entire cohort (Dashboard „Predictors of Disease Progression).

D Urological complications (hemorrhagic events involving gross hematuria or cyst hemorrhages, cyst infections, or flank pain related to cysts) classified by age of first onset as defined in the PROPKD score. Only data from patients who had reached the age of 35 years is included.

E Arterial hypertension classified by age of first onset as defined in the PROPKD score. Only data from patients who had reached the age of 35 years is included.

F Type of urological complication.

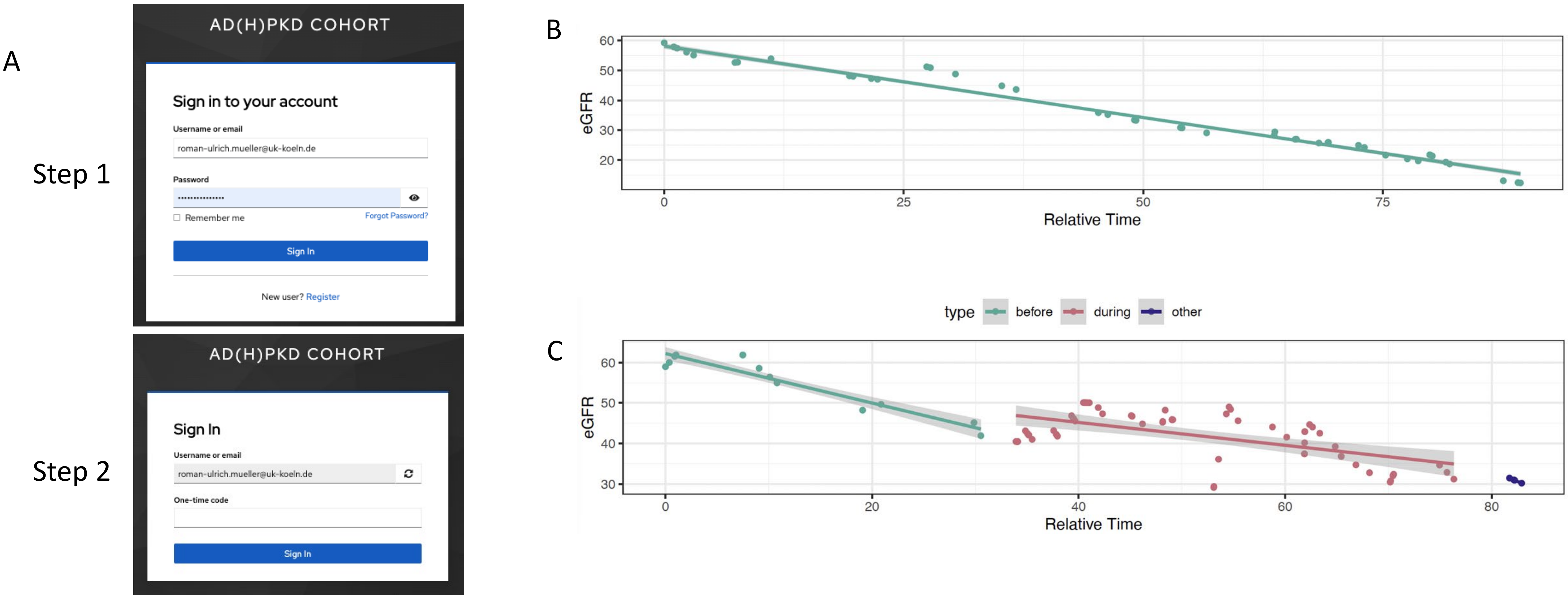

Figure S9: Individual patient-level eGFR trajectories including treatment effects

A MEDA dual authentication log-in using Microsoft Authenticator allows study centers to view individual patient-level data of their patients.

B In this regard, individual eGFR slopes are an important feature, e.g. to assess progression and potential future onset of kidney failure.

C Besides, MEDA can visualize periods on and off treatment (before, during; shown for tolvaptan) to depict individual treatment response. Creatinine values for which treatment status is unknown are shown as „other“.

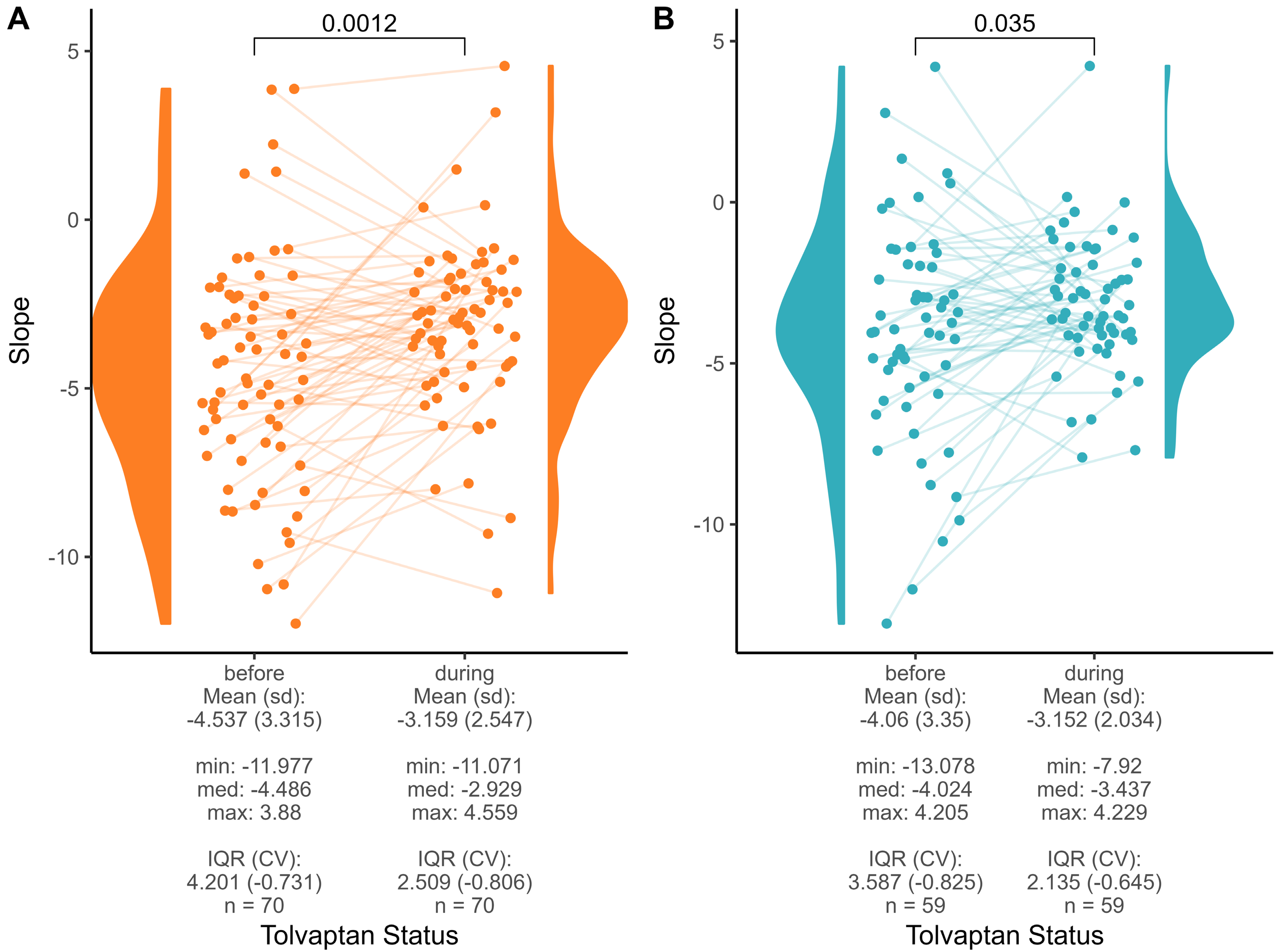

Figure S10: Treatment effect of tolvaptan by sex

A Longitudinal paired analysis of eGFR slopes before and on tolvaptan in male patients only (using all eGFR values available)

B Longitudinal paired analysis of eGFR slopes before and on tolvaptan in female patients only (using all eGFR values available)

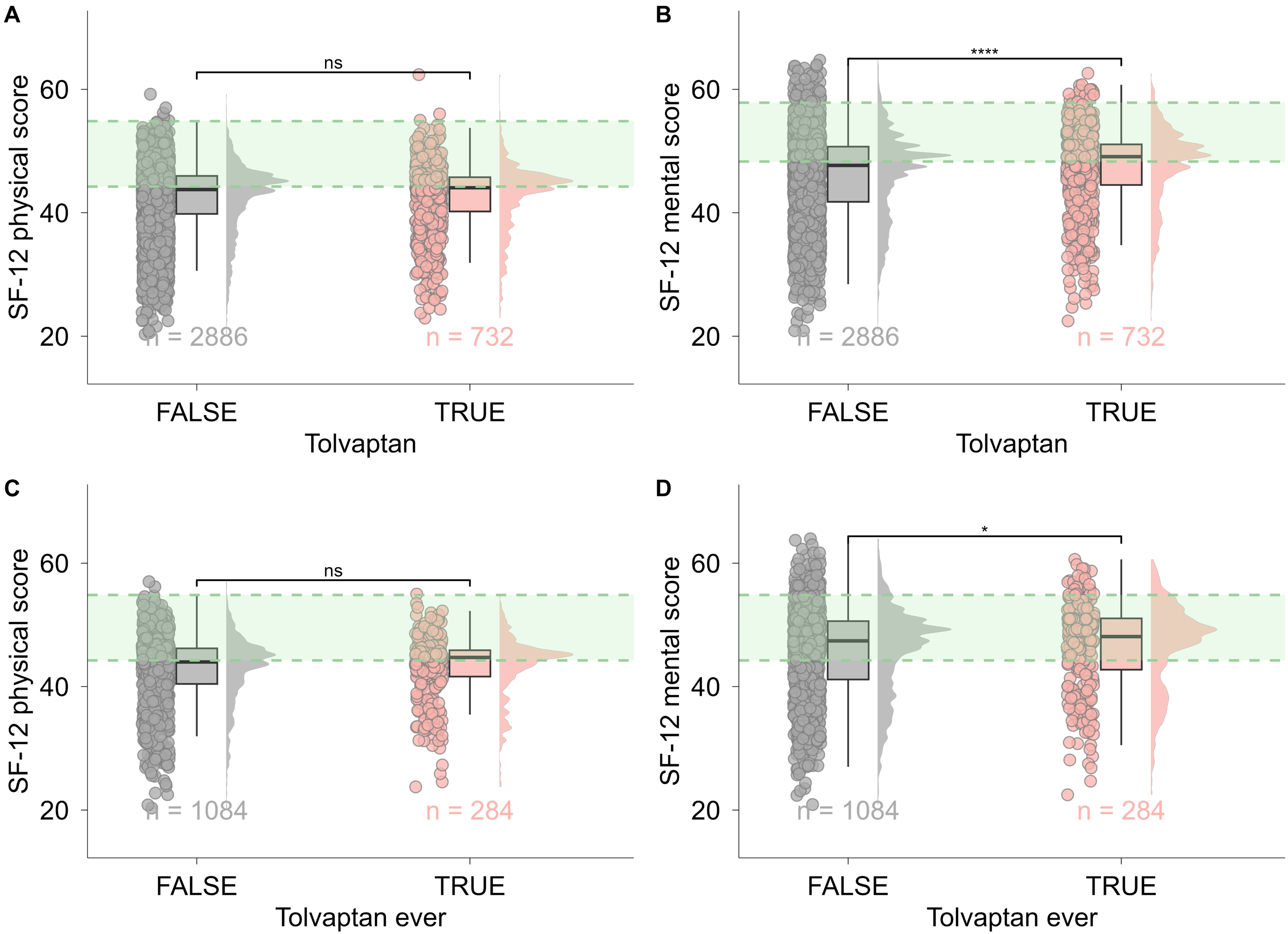

Figure S11: The impact of tolvaptan on quality of life as assessed by the SF-12 questionnaire

A Physical health score using all questionnaires filled while off or on tolvaptan.

B Mental health score using all questionnaires filled while off or on tolvaptan.

C Physical health score comparing the first questionnaire filled by patients never on tolvaptan compared to the first questionnaire on tolvaptan of patients who ever took tolvaptan.

D Mental health score as in C.

Green-shaded area depicts distribution in the normal population.

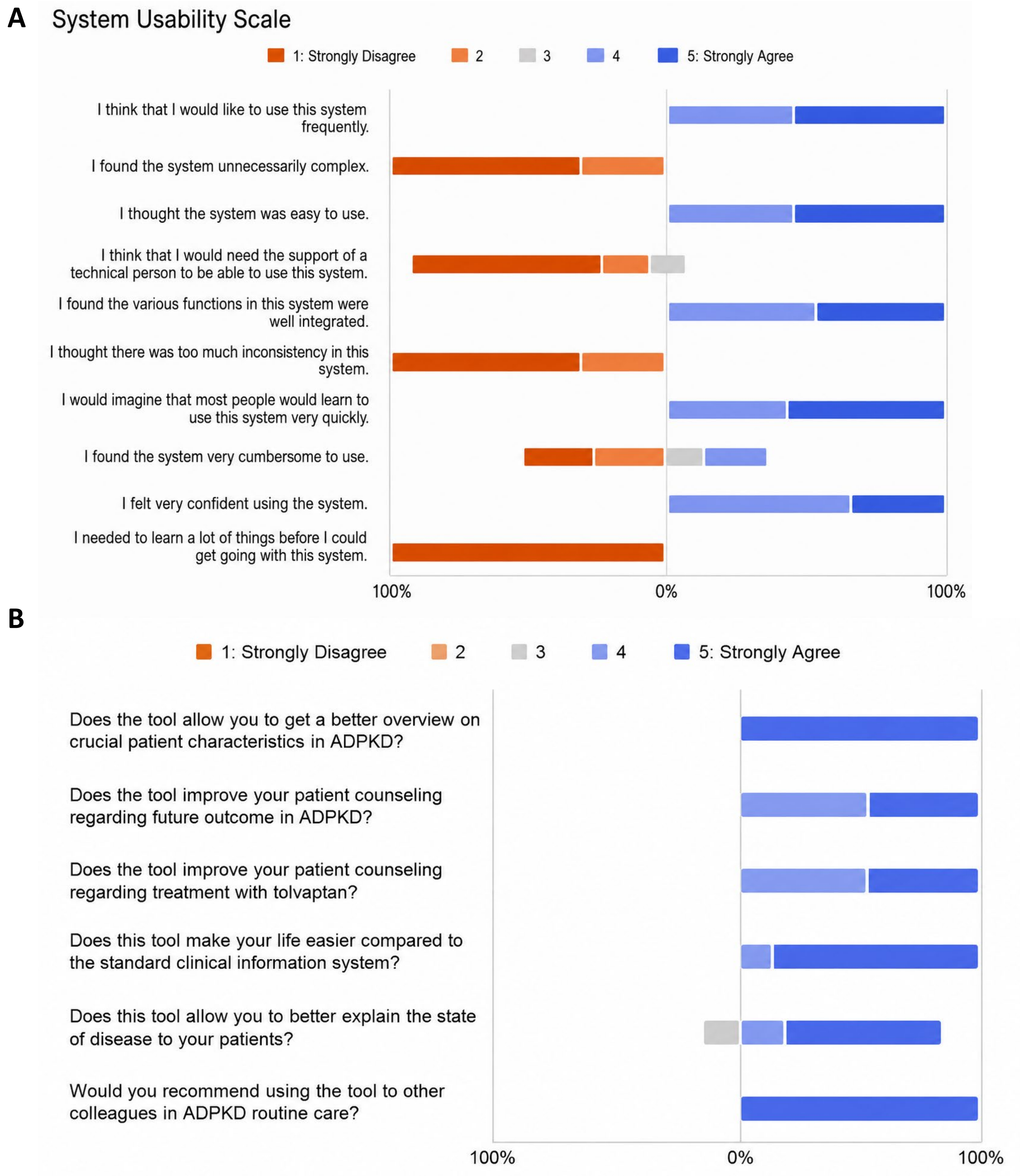

Figure S12: Insights into real-life usability of MEDA-PKD

A System Usability Scale results by tester (7 clinicians experienced in the care for patients with ADPKD), calculation of the overall SUS score based on this assessment results in a value of 90.4 indicating excellent usability (for SUS see Brooke et al. Journal of Usability Studies, Vol. 8, Issue 2, February 2013 pp. 29-40).
B ADPKD-specific questions for the assessment of the tool (same testers as in A).
ChatGPT was used to harmonize the graph design in A / B of this figure (GPT-5.5).

A

1) previous / current treatment with tolvaptan, time periods
   → if yes, urine volume first visit and last visit
   → if yes, urine osmolality first visit and last visit
   → if yes, uptitration to 90/30 mg
2) CKD stage (current / baseline)
3) Mayo Imaging Class
4) body mass index
5) blood pressure (current / baseline)
6) PROPKD score
7) genotype
8) early onset of kidney failure in parents
9) urological symptoms
10) eGFR slope (if applicable: before and on tolvaptan)

B

tester #	% time saving	average % time saving
**#1 - patient 1**	54.97	72.17
**#1 - patient 2**	73.06	
**#1 - patient 3**	88.46	
**#2 - patient 4**	38.33	61.52
**#2 - patient 5**	60.67	
**#2 - patient 6**	85.56	
**#3 - patient 7**	77.96	77.79
**#3 - patient 8**	78.06	
**#3 - patient 9**	77.36	
**#4 - patient 10**	67.96	73.68
**#4 - patient 11**	79.24	
**#4 - patient 12**	73.86	
**#5 - patient 13**	71.22	70.57
**#5 - patient 14**	73.71	
**#5 - patient 15**	66.78	
**#6 - patient 16**	56.73	65.29
**#6 - patient 17**	63.01	
**#6 - patient 18**	76.13	
**#7 - patient 19**	86.06	86.03
**#7 - patient 20**	87.64	
**#7 - patient 21**	84.39	

Figure S13: Quantification of time-savings enabled through MEDA-PKD by real-life testers

A Data points assessed by clinicians in the real-life test to provide counselling towards the indication for tolvaptan

B Time savings to obtain the datapoints from B in the setting of routine care. All testers were physicians experienced in the care for patients with ADPKD and took the timings to obtain this information in 3 independent patient cases (different for each tester). MEDA-PKD was compared to the established clinical information system which had been filled with all information before in the setting of an expert center. The resulting average time to retrieve all information was 634.5 seconds in the routine clinical information setting and 169.4 seconds in MEDA-PKD ($p < 0.001$ using a paired Student's t-test).

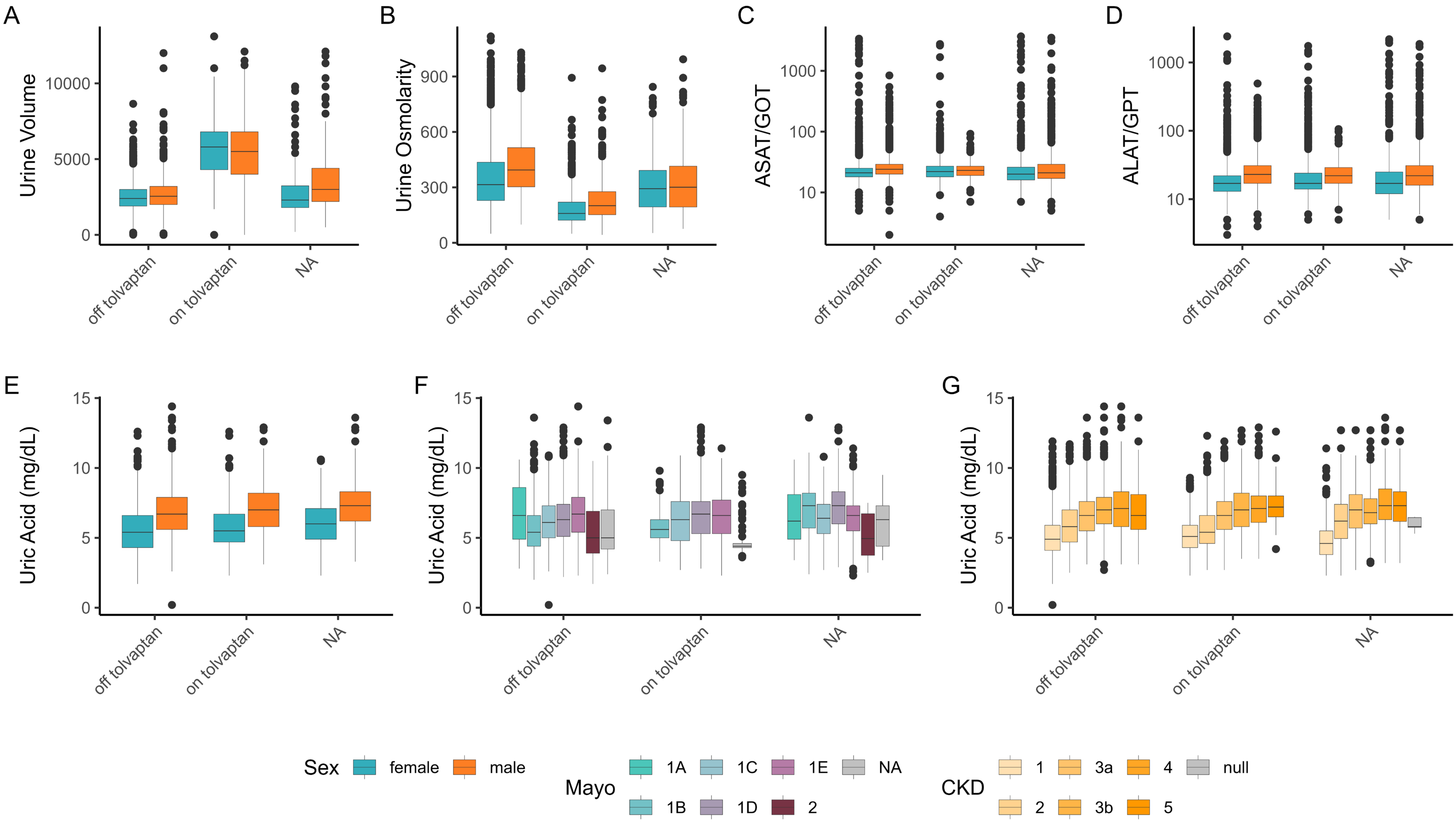

Figure S14: Dashboard „Lab Values“

A-D Standard parameters always shown in the dashboard are urine volume, urine osmolarity, ASAT and ALAT and their relation to tolvaptan status at time of measurement.

E-G The user can select any of the 212 routine lab variables to visualize their relation to sex, Mayo Imaging Class and CKD stage.
